# Demethylase FTO mediates m6A modification of ENST00000619282 to promote apoptosis escape in rheumatoid arthritis and the intervention effect of Xinfeng Capsule

**DOI:** 10.3389/fimmu.2025.1556764

**Published:** 2025-03-13

**Authors:** Fanfan Wang, Jianting Wen, Jian Liu, Ling Xin, Yanyan Fang, Yue Sun, Mingyu He

**Affiliations:** ^1^ The First Affiliated Hospital of Anhui University of Chinese Medicine, Hefei, Anhui, China; ^2^ Department of Rheumatism Immunity, The First Affiliated Hospital of Anhui University of Chinese Medicine, Hefei, Anhui, China; ^3^ Department of Clinical Data Center, The First Affiliated Hospital of Anhui University of Chinese Medicine, Hefei, Anhui, China

**Keywords:** rheumatoid arthritis, Xinfeng Capsule, apoptosis escape, M6A, FTO, ENST00000619282

## Abstract

**Introduction:**

The pathological mechanisms of rheumatoid arthritis (RA) are closely associated with the apoptosis escape of fibroblast-like synoviocytes (FLS). The m6A modification of long non-coding RNAs (lncRNAs) plays a critical regulatory role in RA pathogenesis. Xinfeng Capsule (XFC), a clinically effective traditional Chinese medicine formulation, has been shown to alleviate RA by inhibiting FLS apoptosis escape. However, its molecular mechanisms remain unclear. This study aimed to elucidate the mechanism by which the demethylase FTO promoted FLS apoptosis escape through the m6A modification of lncRNA ENST00000619282 and to reveal the therapeutic targets of XFC in treating RA by intervening in this m6A-dependent pathway.

**Methods:**

A retrospective analysis was conducted on 1603 RA patients using association rule mining and random walk algorithms to evaluate the efficacy of XFC. The proliferation and apoptosis of co-cultured RA-FLS were assessed using CCK-8, flow cytometry (FCM), and molecular biology techniques. Bioinformatics prediction, MeRIP-qPCR, RIP, and RNA pull-down assays were employed to identify the m6A modification sites of ENST00000619282 and their interactions with FTO/YTHDF1. Additionally, FISH, luciferase reporter assays, and rescue experiments were performed to validate the regulatory role of ENST00000619282 and its sponge-like function in RA-FLS. Clinical samples were analyzed to determine the correlation between FTO/YTHDF1/ENST00000619282/Bax/Bcl-2 and immune-inflammatory markers. Furthermore, the binding affinity of XFC active components to NF-κB was assessed through molecular docking.

**Results:**

Retrospective data mining demonstrated that XFC significantly improved immune-inflammatory markers in RA patients. Mechanistically, FTO reduced the m6A modification level of ENST00000619282, enhancing its stability and promoting YTHDF1-dependent expression, which in turn inhibited PUF60 and activated the NF-κB pathway, ultimately leading to FLS apoptosis escape. XFC downregulated FTO, increased the m6A modification of ENST00000619282, blocked the NF-κB signaling, inhibited RA-FLS proliferation, as well as induced their apoptosis. Clinical validation revealed that FTO/YTHDF1/ENST00000619282/Bax/Bcl-2 was closely associated with immune-inflammatory markers in RA patients. After XFC treatment, FTO, ENST00000619282, and Bcl-2 expressions were decreased, while YTHDF1 and Bax expressions were increased (all P<0.05). Molecular docking confirmed that the active components of XFC (calycosin-7-O-beta-D-glucoside, calycosin, and formononetin) exhibited strong binding affinity to NF-κB p65.

**Conclusion:**

FTO promoted FLS apoptosis escape and RA progression by activating the NF-κB pathway through the m6A-dependent ENST00000619282/YTHDF1 axis. XFC inhibited this pathway by modulating FTO-mediated m6A modification, providing a novel RNA epigenetic regulatory strategy for RA treatment.

## Graphical Abstract

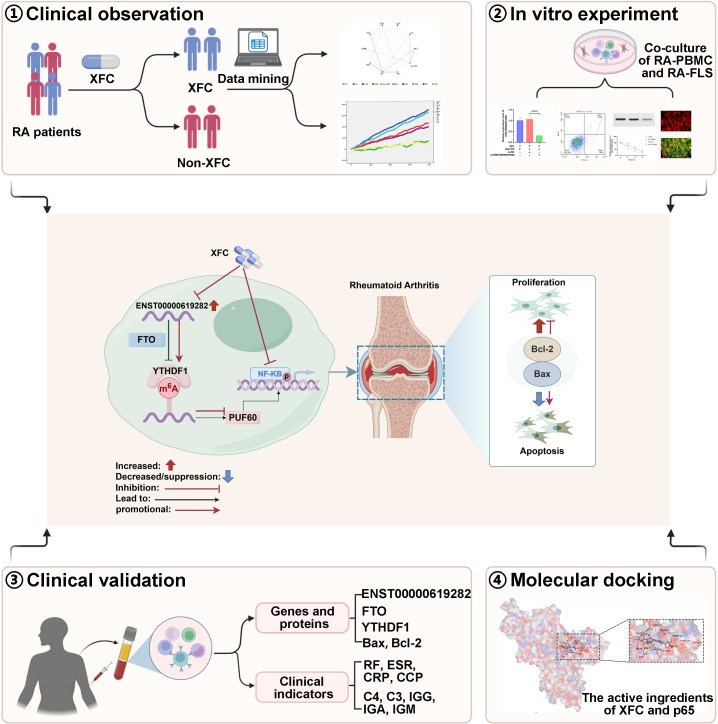

## 1 Introduction

Rheumatoid arthritis (RA) is a chronic, inflammatory, and progressive autoimmune disorder characterized by a high incidence and disability rate. It affects multiple systems and, features a prolonged and lingering disease course, significantly impairing the quality of life of patients (1, 2). The global incidence of RA is approximately 0.3%-1%, with females showing a higher incidence than males, at a ratio of approximately 3:1. However, the exact pathogenesis of RA is still not fully understood at present (3). Currently, the mainstay treatments for RA include nonsteroidal anti-inflammatory drugs (NSAIDs), glucocorticoids, disease-modifying antirheumatic drugs (DMARDs), and biologics. Among these, leflunomide is a classic synthetic DMARD, which can inhibit dihydroorotate dehydrogenase (DHODH) activity, block *de novo* pyrimidine synthesis, and then suppress the proliferation of T cells and B cells, thereby exerting immunomodulatory effects (48). Leflunomide has been widely used in the clinical management of RA, exhibiting favorable efficacy and safety profiles (49). Nevertheless, its clinical application is limited as some patients may experience suboptimal therapeutic responses and side effects such as hepatotoxicity (50). Although anti-RA drugs such as biologics have improved patient outcomes to some extent, approximately 40% of patients still face issues such as poor efficacy, severe side effects, and high treatment costs, which need to be urgently addressed (4). Therefore, an in-depth exploration of the pathogenesis of RA and the discovery of new therapeutic targets and effective drugs hold significant clinical importance and societal value.

RA is typically characterized by pathological features including abnormal proliferation of synovial tissue and inflammatory cell infiltration. Fibroblast-like synoviocytes (FLS), as crucial components of synovial tissue, play a pivotal role in the pathogenesis of RA, exhibiting tumor-like invasive properties, while apoptosis escape is a critical inducer of RA (5). Apoptosis is a programmed cell death process, which is essential for maintaining tissue homeostasis and eliminating damaged or abnormal cells (51). Apoptosis escape refers to the failure of cells to initiate or complete the apoptotic program in response to apoptotic signals, resulting in the survival and accumulation of abnormal cells. In RA, apoptosis escape of synovial cells, particularly FLS, results in their excessive proliferation and enhanced invasiveness, thereby promoting synovial inflammation and joint destruction (52). Furthermore, apoptosis escape may also lead to abnormal survival of immune cells, such as autoreactive T cells and B cells, thus exacerbating autoimmune responses and chronic inflammation (53). The molecular mechanisms underlying apoptosis escape involve dysregulation of multiple signaling pathways, including the death receptor pathway (e.g., Fas/FasL), mitochondrial pathway, and endoplasmic reticulum stress pathway. These dysregulated pathways not only directly contribute to RA pathogenesis but may also amplify inflammatory responses and tissue damage by modulating the release of inflammatory cytokines (such as TNF-α, IL-1β, and IL-6) (54). Multiple *in-vivo* experimental studies have shown that the expression of pro-apoptotic proteins (such as Bax, Fas, caspase-3, and caspase-8) is significantly reduced, while the expression levels of the anti-apoptotic protein Bcl-2 are significantly increased in both adjuvant/collagen-induced arthritis animal models and synovial tissue of RA patients (6, 7). Furthermore, multiple *in-vitro* studies have confirmed this finding, demonstrating apparent apoptotic insufficiency in FLS derived from RA patients and arthritis animal models (8, 9). Preliminary studies from our research group have also indicated that when RA-FLS are stimulated by TNF-α, their apoptosis levels decrease significantly (10).

N6-methyladenosine (m6A) modification, occurring on the sixth N atom of adenine bases, is one of the most common modifications in eukaryotic mRNA and long non-coding RNA (lncRNA). It has been shown that m6A modification participates in the pathogenesis of RA by facilitating precursor RNA splicing, mRNA translation, and enhancing RNA stability, representing a research hotspot and challenge in RNA epigenetics and attracting extensive attention from scholars worldwide (11, 12). Fat mass and obesity-associated protein (FTO) is an m6A demethylase encoded by a gene located on chromosome 16q12.2, first discovered by Hoeven F et al. and believed to be involved in programmed cell apoptosis (13). A previous study has shown that FTO, as a demethylase, enhances the stability of myeloid zinc finger 1 (MZF1) mRNA in an m6A-dependent manner, promotes MZF1 expression, and inhibits lung cancer cell apoptosis (14). Moreover, FTO also mediates the m6A modification of the pro-apoptotic gene Bcl-2/adenovirus E1B 19kDa-interacting protein 3 (BNIP3), reduces BNIP3 stability, and induces its degradation, thereby inhibiting breast cancer cell apoptosis, suggesting that regulating the level of cell apoptosis may be a potential target for FTO to exert its biological functions (15). Additionally, it has been evidenced that abnormal expression of FTO in peripheral blood mononuclear cells (PBMCs) from RA patients is a risk factor for RA and is closely related to disease activity indicators [such as DAS28-erythrocyte sedimentation rate (ESR), DAS28-C-reactive protein (CRP), complement C3 (C3), immunoglobulin G (IGG), platelet-lymphocyte ratio (PLR), and lymphocyte-monocyte ratio (LMR)] (16).

LncRNA is a class of RNAs with longer than 200 nucleotides in length and does not encode proteins, which can regulate gene expression at epigenetic, transcriptional, and post-transcriptional levels and participate in multiple biological processes such as proliferation, apoptosis, and development in various diseases, including RA (17, 18). Our previous high-throughput sequencing of the whole transcriptome, with a p-value < 0.05 and a fold change > 2 as criteria, identified 341 differentially expressed lncRNAs in RA patients. GO and KEGG analyses screened the key lncRNA ENST00000619282 involved in RA cell apoptosis escape. After expanding the clinical sample size, it’s revealed that ENST00000619282 expression is elevated in RA patients and closely related to disease activity indicators such as ESR, CRP, and DAS28 scores (19). ENST00000619282 also participates in the cell apoptosis escape mechanism by regulating apoptosis-related proteins (10). However, its precise mechanism remains unclear and deserves further exploration.

The activation of the NF-κB signaling pathway plays a pivotal role in the inflammatory and pathological processes of RA. As has been evidenced previously, NF-κB is an inducible transcription factor involved in immune inflammatory responses, cell cycle progression, inhibition of cell apoptosis, and cell adhesion, thereby promoting chronic inflammatory responses (20, 21). Studies have shown that abnormal activation of NF-κB not only directly contributes to the inflammatory response in RA synovial cells but also promotes apoptosis escape by regulating the expression of apoptosis-related genes, further exacerbating the pathological progression of RA (55–58). Therefore, the NF-κB signaling pathway plays a crucial role in both inflammation and apoptosis escape in RA, representing a significant potential therapeutic target for RA treatment.

RA belongs to the category of “Bi Syndrome” in Traditional Chinese Medicine (TCM), and “spleen deficiency with excessive dampness” is considered the pathogenic mechanism of RA in TCM, which is analogous to the molecular biological mechanism of apoptotic escape in RA cells. Based on long-term theoretical research and clinical practice, we have proposed the important academic viewpoints of “Bi Syndrome arising from spleen deficiency” and “treating RA from the spleen” and developed Xinfeng Capsule (XFC, WanYaoZhiZi: Z20050062, Invention Patent No.: ZL201310011369.8). XFC is developed by the First Affiliated Hospital of Anhui University of Traditional Chinese Medicine, comprising *Astragalus mongholicus* Bunge (http://mpns.kew.org), *Coix lacryma-jobi* L. (http://mpns.kew.org), *Tripterygium wilfordii* Hook. f. (http://mpns.kew.org), and *Scolopendra subspinipes mutilans* L.Koch (https://db.ouryao.com), at a ratio of 20:20:10:1. Previous studies have demonstrated that high-performance liquid chromatography (HPLC) has established a fingerprint, confirming that XFC possesses excellent production technology and meets stringent quality standards (22). As indicated by our team’s earlier large-sample, multicenter, randomized, double-blind, double-dummy clinical RCT trial (ClinicalTrials.gov Identifier: NCT01774877), XFC can significantly alleviate joint symptoms, laboratory indicators, and the quality of life of RA patients, showing superior overall efficacy to leflunomide (23). Meta-analysis results indicated that compared to leflunomide, XFC can significantly alleviate joint pain, swelling, and duration of morning stiffness, as well as reduce ESR and CRP levels and anti-cyclic citrullinated peptide (CCP) antibody levels (46). These studies provide high-level evidence-based medical evidence for the effectiveness of XFC in treating RA. Preliminary clinical studies have shown that XFC promotes CD4^+^T cell apoptosis in RA patients by upregulating the expression of pro-apoptotic proteins (Fas, FasL, caspase-8, and caspase-3) and downregulating the expression of anti-apoptotic protein Bcl-2 (24). Animal experiments have indicated that XFC remarkably reduces secondary foot swelling and the multiple arthritis index, improves joint histopathological damage, inhibits the NF-κB pathway activation, and promotes synovial cell apoptosis in AA rats (47). Additionally, XFC-containing serum can effectively promote TNF-α-induced apoptosis of RA-FLS, inhibit abnormal proliferation of RA-FLS, and alleviate RA conditions by regulating lncRNA MAPKAPK5-AS1 (25). Therefore, clinical, animal, and cellular experiments have all demonstrated that XFC can inhibit RA cell apoptosis escape, which deserves further in-depth exploration.

To further elucidate the potential mechanism of XFC in inhibiting cell apoptosis escape, this study adopted a comprehensive multi-level strategy, integrating retrospective data mining, cellular experiments, bioinformatics predictions, clinical validation, and molecular docking. We proposed a hypothesis that FTO could regulate the m6A modification of ENST00000619282, activate the NF-κB signaling pathway, and mediate cell apoptosis escape to participate in RA pathogenesis. XFC exerted its therapeutic effect by regulating FTO-mediated m6A modification of ENST00000619282 to inhibit RA cell apoptosis escape. This study aimed to use PBMCs and FLS coculture cells from RA patients as the research objects, with FTO regulation of the m6A modification of ENST00000619282 as the main line, cell apoptosis escape as the target, and XFC as the intervention measure. A series of experimental methods [such as methylated RNA immunoprecipitation-quantitative polymerase chain reaction (MeRIP-qPCR), RNA pull-down, and methylation site mutation] were employed in this study to elucidate the mechanism by which FTO regulated the m6A modification of ENST00000619282 to mediate RA cell apoptosis escape and the interventional role of XFC from the perspective of epigenetic modification, providing new targets and scientific evidence for XFC treatment of RA.

## 2 Materials and methods

### 2.1 Clinical data retrieval

All clinical data of 1,603 discharged RA patients were collected from the electronic medical record system of the Rheumatology Department of the First Affiliated Hospital of Anhui University of Chinese Medicine. All RA patients met the RA diagnostic criteria proposed by the American College of Rheumatology (ACR) in conjunction with the European League Against Rheumatism (EULAR) in 2010 (26). The included study indicators were as follows: ESR, high-sensitivity C-reactive protein (Hs-CRP), rheumatoid factor (RF), CCP, immunoglobulin A (IGA), IGG, immunoglobulin M (IGM), C3, complement C4 (C4), alanine aminotransferase (ALT), and aspartate aminotransferase (AST). This study received approval from the Ethics Committee of the First Affiliated Hospital of Anhui University of Chinese Medicine (Ethical Approval Number: 2023AH-28). A retrospective data mining approach was adopted in this study, which fully protected patient privacy without affecting their treatment plans. The ethics committee waived the requirement for informed consent. Additionally, all data were rigorously de-identified (removing personal information such as names and ID numbers) to ensure patient confidentiality, and each patient was assigned a unique code to maintain anonymity. Data were independently entered by two researchers and cross-checked to ensure accuracy. All data were stored on the hospital’s encrypted internal servers and were only accessible to authorized researchers only.

### 2.2 Cell culture

PBMCs were isolated from 5 mL of venous blood collected from RA patients using EDTA anticoagulant tubes. The blood was mixed with an equal volume of phosphate-buffered saline (PBS) and subjected to Ficoll gradient centrifugation to isolate PBMCs, which were then stored for subsequent use. RA-FLS cells (Beijing Beina Chuanglian Biotechnology Research Institute, with STR authentication reports) were cultured in DMEM medium supplemented with 15% fetal bovine serum, 100 U/mL penicillin, and 100 mg/mL streptomycin. The cells were maintained in a 5% CO_2_ incubator at 37°C. The medium was replaced three times a week, and when cells reached 80% confluence, they were passaged at a ratio of 1:1 or 1:2. For co-culture experiments, RA-FLS cells were digested, centrifuged at 1000 rpm for 15 minutes, and resuspended in DMEM medium. After adjusting cell concentration, the RA-FLS cells were seeded in the lower chamber of a Transwell system. RA-PBMCs were then seeded in the upper chamber of the Transwell. Following co-culture, RA-FLS cells were collected for subsequent experiments.

### 2.3 Cell transfection

pcDNA3.1-FTO, pcDNA3.1-LncRNA ENST00000619282, pcDNA3.1-PUF60 overexpression vectors, and siRNA were constructed and transfected into RA-FLS using Lipofectamine 2000. Cells transfected with different concentrations of pcDNA3.1-FTO, pcDNA3.1-LncRNA ENST00000619282, pcDNA3.1-PUF60, and siRNA were collected at 24h, 48h, and 72h time points. Reverse transcription-qPCR (RT-qPCR) was performed to detect the expression levels of FTO, ENST00000619282, and PUF60, allowing for the selection of the optimal transfection time and concentration for subsequent experiments.

### 2.4 Colorimetric detection of total m6A levels

Total m6A levels in extracted RNA were detected using an m6A RNA methylation quantification kit following the manufacturer’s instructions. Briefly, total RNA was bound to wells using an RNA high-binding solution. m6A was detected utilizing capture and detection antibodies. After adding a chromogenic reagent, the m6A level was quantified by measuring the optical density (OD) value at 450 nm.

### 2.5 Detection of ENST00000619282 m6A levels using MeRIP-qPCR

Total RNA was extracted from RA-FLS, and added with premixed m6A antibodies on immunomagnetic beads. The m6A immunomagnetic beads were enriched using a magnetic rack, and the enriched RNA-antibody complexes were digested with protease. The primers for ENST00000619282 were designed, and subsequent operations were performed according to qPCR procedures to detect the m6A level of ENST00000619282 in RA-FLS.

### 2.6 RNA extraction and RT-qPCR

Total RNA was isolated from RA-FLSs using TRIzol reagent as per the manufacturer’s protocol. Subsequently, RNA was reverse-transcribed into cDNA using a reverse transcription kit. RT-qPCR analysis was conducted using TB Green™ Premix ExTaq™ II (TaKaRa). The reaction conditions were as follows: initial denaturation at 95°C for 1 minute, followed by 40 cycles of denaturation at 95°C for 20 seconds, and annealing/extension at 60°C for 1 minute. All primers were synthesized by Sangon Biotech Co., Ltd. (Shanghai, China). Data were analyzed using the 2-ΔΔCt method, with β-actin expression as an internal reference for relative quantification. The primer sequences for each detection indicator are shown in **Table 1**.

**Table 1:** RT-qPCR real-time primer sequences.

Gene	Amplicon Size (bp)	Forward primer (5’→3’)	Reverse primer (5’→3’)
Hu-β-actin	96	CCCTGGAGAAGAGCTACGAG	GGAAGGAAGGCTGGAAGAGT
Hu-FTO	148	AGACACCTGGTTTGGCGATA	GTTCCTGTTGAGCACTCTGC
Hu-YTHDF1	143	TTGTGGAATGAGGGACCGTT	AAGCGTCTGCTCTCTAGGAC
hsa-ENST00000619282.1	183	TCAGGAATCGGAGGAAGTGG	CCTTGCCTCTGAGGGTCTTA
FTO-homo-124	/	GCAGCUGAAAUAUCCUAAATT	UUUAGGAUAUUUCAGCUGCTT
FTO-homo-352	/	GCCAGUGAAAGGGUCUAAUTT	AUUAGACCCUUUCACUGGCTT
FTO-homo-722	/	GUGGCAGUGUACAGUUAUATT	UAUAACUGUACACUGCCACTT
YTHDF1–homo-1025	/	CACCGTCCACCCATAAAGCATAACATTTCA AGAGAATGTTATGCTTTATGGGTGGATTTTTTG	GATCCAAAAAATCCACCCATAAAGCATAACATTCTCTTG AAATGTTATGCTTTATGGGTGGAC
YTHDF1–homo-1403	/	CACCGCGGGCGTGTGTTCATCATCAATTCAAGA GATTGATGATGAACACACGCCCGTTTTTTG	GATCCAAAAAACGGGCGTGTGTTCATCATCAATCTCTT GAATTGATGATGAACACACGCCCGC
YTHDF1–homo-1715	/	CACCGCAGGCTGGAGAATAACGACAATTCAAGAGA TTGTCGTTATTCTCCAGCCTGTTTTTTG	GATCCAAAAAACAGGCTGGAGAATAACGACAATCTC TTGAATTGTCGTTATTCTCCAGCCTGC
Hu-PUF60	128	AATGGAAACCTCCACAGGGC	GGCGTACTTCTTGGCCTTCT

### 2.7 Western blot

Cell pellets were collected, and 100 μL of RIPA lysis buffer (containing 1 mM PMSF) was added per 6-well plate. The cells were lysed on ice for 30 minutes, followed by centrifugation at 12,000 rpm for 15 minutes, with the supernatant collected as total protein. After that, 5× SDS-PAGE loading buffer was added at a 1:4 ratio, and the mixture was heated in a boiling water bath for 10 minutes and then cooled to room temperature. The denatured proteins were loaded onto a sodium dodecyl sulfate-polyacrylamide gel electrophoresis (SDS-PAGE) gel, subjected to electrophoresis, and then transferred to polyvinylidene fluoride (PVDF) membranes (pre-activated with methanol for 3 minutes). The membranes were blocked with 5% skim milk at room temperature for 2 hours, followed by incubation with primary antibodies overnight at 4°C and then three PBST washes (10 minutes each). Next, secondary antibodies (diluted 1:10,000) were added and incubated at room temperature for 2 hours, followed by three additional PBST washes (10 minutes each). Protein levels were detected using an enhanced chemiluminescence detection kit (Thermo Fisher, Waltham, MA, USA), and quantitative analysis was performed using ImageJ software. All experiments were repeated three times.

### 2.8 Cell counting kit-8 assay

During recovery, the co-cultured RA-FLS (1×10^5^/mL) were plated in 96-well plates. After 1 hour of incubation, 100 μL of CCK-8 was added to each well, and the edge wells were filled with sterile PBS. The plated cells were cultured overnight in an incubator at 37°C with 5% CO_2_. The transfection group underwent transfection, and 6 hours later, a complete medium containing 10 ng/mL TNF-α and drug-containing serum was added to the cell group. After 48 hours of culture, 10 μL of CCK-8 was added to each well, followed by an incubation for 1 hour. The OD value of each well was measured at 450 nm using a microplate reader. Blank wells (containing medium and CCK-8) were also set up as the control.

### 2.9 Flow cytometry

The co-cultured RA-FLS cells were digested using trypsin without EDTA, and 0.5-1 × 10^6^ cells were collected. After centrifugation at 1500 rpm for 3 minutes, the supernatant was discarded. The cells were washed twice with pre-chilled PBS (1500 rpm, 3 minutes each) and then resuspended in 100 μL of 1× binding buffer. Subsequently, 5 μL of FITC was added for incubation in the dark for 15 minutes, followed by the addition of 5 μL of PI for incubation in the dark at room temperature for 5 minutes. Data were acquired using a flow cytometer, and the apoptosis rate was analyzed using NovoExpress software. The total percentage of cells in quadrant 2 (late apoptosis) and quadrant 4 (early apoptosis) was calculated as the apoptosis rate.

### 2.10 Actinomycin D experiment

After digestion, centrifugation, and resuspension, the cells were plated in 6-well plates. Cells were allocated into the si-NC group, FTO siRNA group, pcDNA3.1-NC group, and pcDNA3.1-FTO group. The cells were treated with 5 μg/mL actinomycin D for 0, 2, 4, and 8 hours, respectively. Cell RNA was extracted, and examined for the expression of ENST00000619282 using RT-qPCR.

### 2.11 RNA pull-down assay

Biotin-labeled RNA probes (sense and antisense strands) for ENST00000619282 were synthesized and sequence-verified by Guangzhou Boxin Biotechnology Co., Ltd. (Guangdong, China). The probes were denatured (90°C water bath for 2 minutes) and renatured (ice bath for 2 minutes) to form stable secondary structures. Streptavidin magnetic beads were washed with 1× TES buffer and blocked with yeast tRNA and bovine serum albumin (BSA) to reduce non-specific binding. The biotin-labeled RNA probes were incubated with the beads in 2× TES buffer for 30 minutes to form probe-bead complexes. Next, RA-FLS cell lysates (containing protease inhibitors) were added to the probe-bead complexes and rotated at room temperature for 2 hours to ensure sufficient RNA-protein binding. After that, the complexes were washed four times with ice-cold NT2 buffer (containing 0.1% NP-40) to remove unbound proteins and improve the binding specificity. RNA-binding proteins were eluted using protein elution buffer (containing DTT) at 37°C for 2 hours. The eluted proteins were separated by SDS-PAGE, and silver staining was used to visualize differential bands. Candidate proteins were identified by mass spectrometry.

### 2.12 RNA immunoprecipitation combined with quantitative PCR

A total of 2 × 10^7^ RA-FLS cells were collected, washed with PBS, and lysed in polysome lysis buffer (containing protease inhibitors and RNase inhibitors) on ice for 10 minutes. After brief freeze-thaw cycles at -80°C, the lysates were centrifuged, with the supernatant collected. For DNA removal, DNase I (20 U) was added to the lysate, followed by incubation at 37°C for 10 minutes, and the reaction was terminated with EDTA/EGTA. The lysate was centrifuged at 16,100 × g for 10 minutes, with the supernatant collected. The lysate was allocated into three groups: IP group (0.8 mL), IgG control group (0.8 mL), and Input group (0.1 mL). The IP group was incubated with 5 μg of PUF60 antibody overnight at 4°C, followed by the addition of pre-equilibrated protein A/G magnetic beads and further incubation at 4°C for 1 hour. The beads were washed thrice with polysome washing buffer 1 and twice with buffer 2, and RNA was eluted using elution buffer. RNA was extracted using TRIzol, precipitated with isopropanol, washed with 80% ethanol, dissolved in RNase-free water, and stored at -80°C. cDNA was synthesized using the NovoScript^®^ cDNA Synthesis Kit (Novoprotein, Cat# E041-01B) after incubation at 42°C for 20 minutes, and the reaction system contained 1-10 μg RNA, Random N6 primers, and reverse transcriptase. Primer sequences for the target gene ENST00000619282.1 were as follows: F: GGCTCTTATGCAAGCCCTTC, R: GGTTTGTTTCCAGTGCCCAT (amplification product: 160 bp). The reaction system consisted of 10 μL of 2× SYBR SuperMix, 1 μL each of forward and reverse primers and 2 μL of cDNA. The reaction conditions were: pre-denaturation at 95°C for 1 minute, followed by 40 cycles of 95°C for 20 seconds and 60°C for 45 seconds. Data were collected in triplicate, and Ct values were recorded (**Table 2**).

**Table 2:** Raw Ct values for the Input, IP, and IgG groups in the RIP-qPCR experiment.

Group	Repeat the 1 Ct values	Repeat the 2 Ct values	Repeat the 3 Ct values	mean ± SD
Input	25.56	25.27	25.26	25.36 ± 0.16
IP	29.19	28.97	29.20	29.12 ± 0.12
IgG	32.34	32.72	32.69	32.58 ± 0.20

### 2.13 RNA fluorescence *in situ* hybridization

Cells were adjusted to a density of 1×10^5^ cells/mL and cultured for 24 hours. After cell digestion with protease K working solution at 37°C for 5 minutes, the cells on the slides were circled with an immunohistochemical pen, and an appropriate amount of digoxin-labeled ENST00000619282 probe was added. The cells were incubated at 55°C for 60 minutes and then transferred to 37°C for hybridization for 3 hours or overnight. The PBS solution was removed, and the primary antibodies (mouse anti-digoxin and rabbit anti-PUF60) were added for incubation at room temperature for 1 hour. After that, goat anti-mouse FITC fluorescent secondary antibody and goat anti-rabbit Cy3 fluorescent secondary antibody were added. A blank control was set up simultaneously. DAPI staining mounting medium was immediately added and mounted. The co-localization of ENST00000619282 and PUF60 in the cells was observed under a fluorescence microscope.

### 2.14 Preparation of XFC drug-containing serum

A total of 30 male Sprague-Dawley (SD) rats were randomly allocated into a normal serum group (n = 10) and an XFC drug-containing serum group (n = 20). According to previous studies, rats in the XFC drug-containing serum group were gavaged with an XFC suspension at 0.648 g/100g/d (27); rats in the normal serum group were gavaged with 2 mL/100g/d of saline for 7 consecutive days. One hour after the last administration, the rats were anesthetized using pentobarbital sodium (50 mg/kg). Rat blood was then collected from the abdominal aorta into procoagulation tubes, centrifuged at 3000 r/min for 10 minutes to obtain serum, and then incubated at 56°C in a constant temperature water bath for 30 minutes for inactivation. After filtering through a 0.22 μm membrane, the serum was stored at -80°C for future use. All procedures involving animals in this study were approved by the Animal Ethics Committee of Anhui University of Chinese Medicine (AHUCM-rats-2023,020).

### 2.15 Clinical sample validation

First, 5 mL of venous blood was collected from 30 RA patients. PBMCs were extracted using the Ficoll gradient centrifugation method, and the expression levels of FTO, ENST00000619282, YTHDF1, Bax, and Bcl-2 in PBMCs from RA patients were detected using RT-qPCR. Our clinical sample collection was conducted following the guidelines for biomedical research involving human subjects in the Helsinki Declaration. This study was approved by the Research Ethics Committee of the First Affiliated Hospital of Anhui University of Traditional Chinese Medicine (Ethical Approval Number: 2023AH-28).

### 2.16 Association rules

The use of XFC treatment was designated as “T” and non-use as “F”. After treatment, improvement in indicators was designated as “T”, and no improvement as “F”. The Apriori module in IBM SPSS Modeler 18.0 software was used to perform association rule analysis between the use of XFC and the improvement of indicators. Moreover, the support, confidence, and lift were calculated, and the specific calculation formulas referred to previous studies (27).

### 2.17 Random walk analysis

The random walk model was employed to assess changes in immune-inflammatory indicators among RA patients following XFC intervention, aiming to establish a long-term relationship between disease improvement and the XFC intervention received by patients. The specific calculation formula for the random walk analysis is detailed in previous research (28).

### 2.18 Molecular docking

The mol2 files of the active ingredients of XFC (calycosin-7-O-beta-D-glucoside, calycosin, and formononetin) were downloaded from the TCSMP database. The PDB file of P65 was downloaded from the PDB database. These files were then imported into CB-Dock2 for molecular docking analysis (29).

### 2.19 Statistical analysis

GraphPad Prism 8.1 software was employed for statistical analysis. Initially, the distribution of the data was assessed through a normality test. Based on the normality of the data, different descriptive statistical methods were employed to present the data. Specifically, continuous variables with normal distribution were presented as the median or mean ± standard deviation (SD), while those with non-normal distribution were presented as the median and interquartile range (IQR). For comparisons between groups, appropriate test methods were selected based on the normality of the variables. Briefly, the Welch’s t-test or analysis of variance (ANOVA) was used for comparisons of continuous variables with normal distribution, while the Wilcoxon rank-sum test or Kruskal-Wallis test was used for those with non-normal distribution. Additionally, all multiple comparisons were corrected to control the risk of false positives due to multiple comparisons. In short, pairwise comparisons following ANOVA or Kruskal-Wallis tests were adjusted using the Bonferroni method, while correlation analyses were corrected using the False Discovery Rate (FDR) method. Spearman correlation analysis was performed to assess the relationships between variables, and *p*-values were adjusted using the FDR method. In all statistical analyses, a corrected *p*-value of less than 0.05 was considered statistically significant.

## 3 Results

### 3.1 The effects of XFC treatment on immuno-inflammatory indicators are evaluated by association rules and random walking analysis

**Figure 1:**
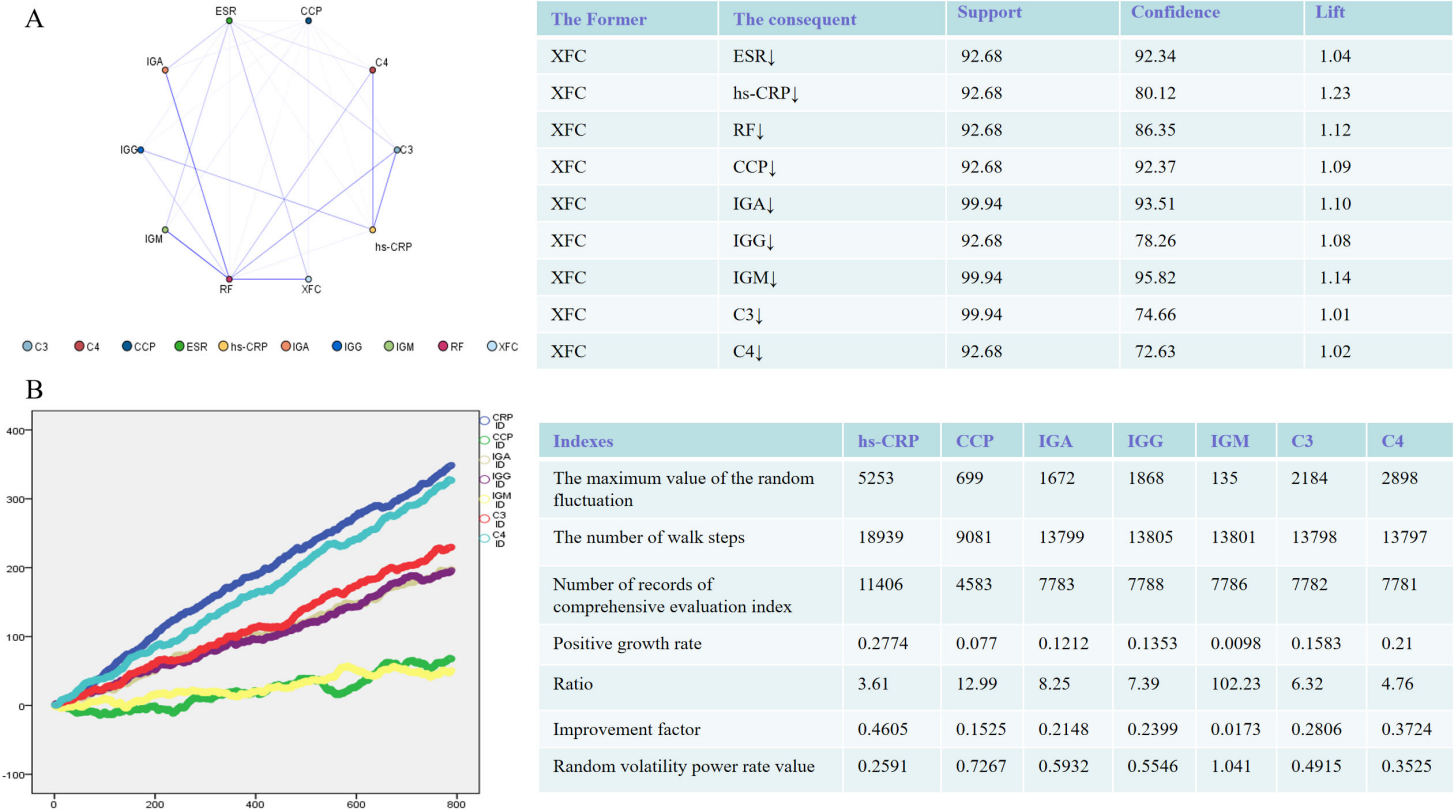
Immune-inflammatory indicators in RA Patients (n = 1603) treated with XFC were evaluated through association rule and random walk analysis. **(A)** Association rule analysis to examine the correlation between XFC and indicator improvement. **(B)** Random walk model demonstrating the long-term association between XFC application and indicator improvement.

To gain a deeper understanding of the efficacy of XFC treatment on immuno-inflammatory indicators, we analyzed the association rules within the dataset to examine the connections between XFC treatment and these indicators. The results revealed that the improvement of immuno-inflammatory indicators following XFC treatment was identified with high confidence, support, and lift values (**Figure 1A**). Additionally, the random walking analysis demonstrated a significant long-range association between XFC treatment and immuno-inflammatory indicators. Briefly, a longer duration of XFC application suggested a more pronounced improvement in these indicators (**Figure 1B**). Overall, these findings indicated that XFC treatment in RA patients was the factor most strongly correlated with improved immuno-inflammatory indicators.

### 3.2 FTO mediates m6A modification of ENST00000619282, with elevated expression in RA

Previous studies have shown that RA-PBMCs can promote the proliferation of RA-FLSs and exacerbate their inflammatory responses. Additionally, it has been found that when the ratio of RA-PBMCs to RA-FLSs is 2.5:1, the proliferative response of RA-FLSs is the most significant, reaching a peak after 48 hours of culture (30). Based on this finding, a ratio of 2.5:1 was established as the standard for the model group in this study for consistency and validity in subsequent experiments.

**Figure 2:**
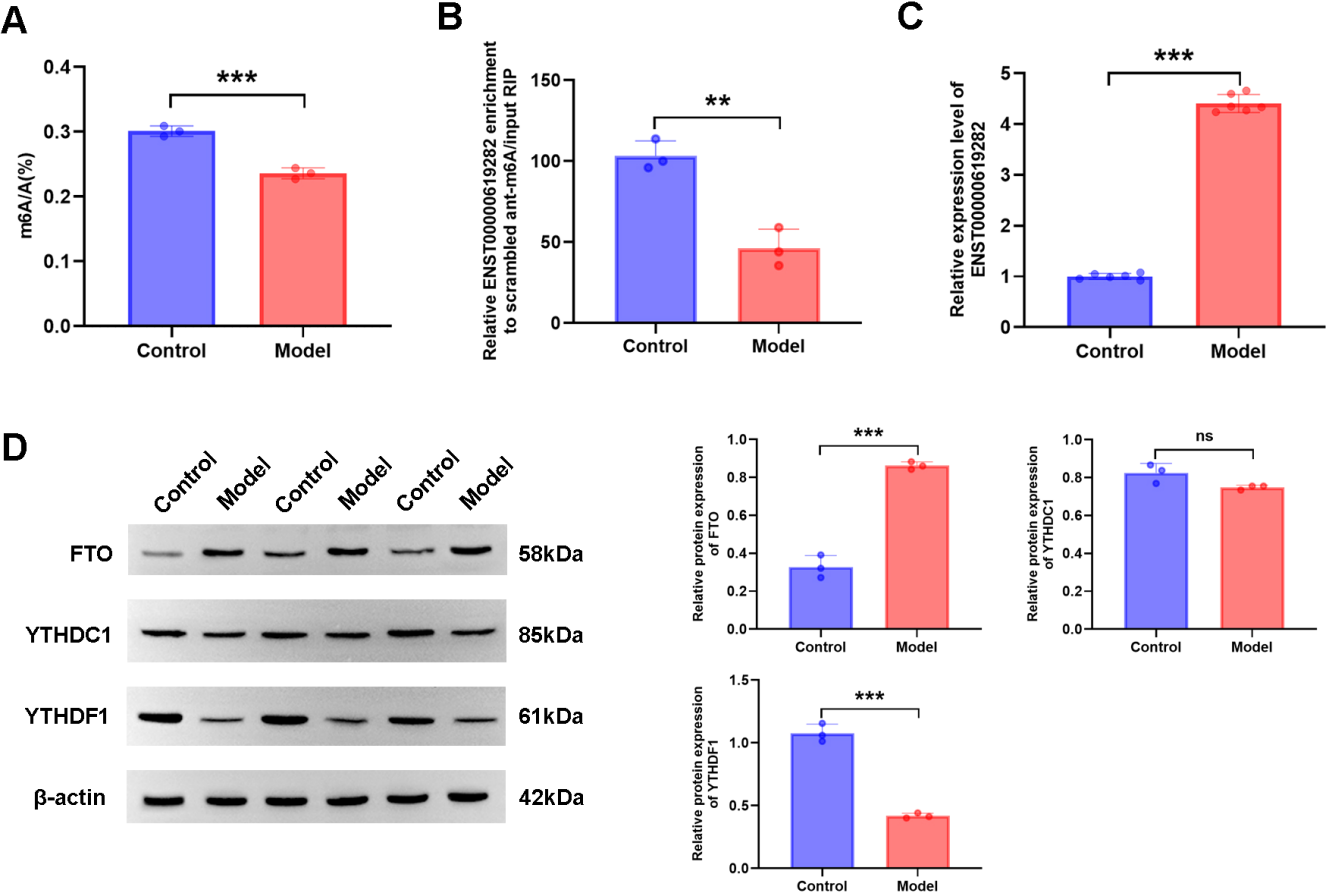
FTO mediates m6A modification of ENST00000619282. **(A)** Decreased total m6A level in the model group. **(B)** Reduced m6A level of ENST00000619282 in the model group. **(C)** Elevated level of ENST00000619282 in the model group. **(D)** Protein bands and relative expression levels of FTO, YTHDC1, and YTHDF1. **p < 0.01, ***p < 0.001. Control group: RA-FLS. Model group: co-culture of RA-PBMC and RA-FLS. ns, no significant.

Next, we focused on changes in m6A modification and the expression of related genes in the model group. According to experimental results, compared to those in the control group, both the total m6A level and the m6A modification level of ENST00000619282 were significantly decreased in the model group (**Figures 2A, B**), while the expression level of ENST00000619282 was notably increased (**Figure 2C**). The upstream methylation regulatory mechanisms were further explored. Through WB technology, the expression of three key proteins (the demethylase FTO, and the m6A reader proteins YTHDC1 and YTHDF1) was examined. As indicated by the results, the model group showed remarkably increased protein expression levels of FTO (**Figure 2D**), suggesting that FTO may play an important role in demethylation in the RA environment. However, YTHDF1 protein level was decreased (**Figure 2D**), indicating that it’s potentially related to its limited function in RNA degradation or translational regulation following m6A modification; in contrast, the protein level of YTHDC1 did not change significantly (**Figure 2D**). These findings revealed that FTO may promote the upregulation of ENST00000619282 expression by mediating its m6A modification in RA, providing a new perspective and potential intervention target for understanding RA pathogenesis.

### 3.3 FTO promotes the proliferation and apoptosis escape of co-cultured RA-FLS by downregulating m6A modification of ENST00000619282

**Figure 3:**
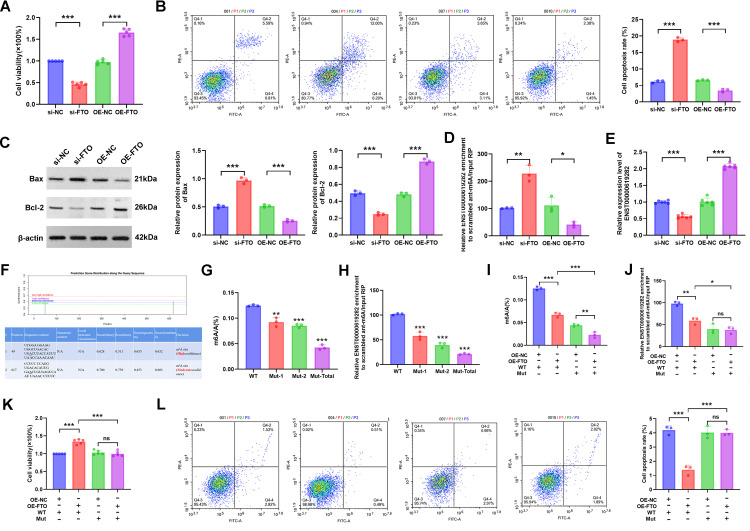
FTO promotes co-cultured RA-FLS proliferation and apoptosis escape by downregulating the m6A modification of ENST00000619282. **(A)** The effect of FTO knockdown/overexpression on RA-FLS cell proliferation. **(B)** The effect of FTO knockdown/overexpression on RA-FLS cell apoptosis. **(C)** The impact of FTO knockdown/overexpression on apoptotic proteins (Bax and Bcl-2). **(D)** The influence of FTO knockdown/overexpression on the m6A modification of ENST00000619282. **(E)** The effect of FTO knockdown/overexpression on ENST00000619282 expression. **(F)** Bioinformatics prediction of two m6A methylation mutation sites on the ENST00000619282 sequence. **(G, H)** The impact of mutated methylation sites on total m6A and ENST00000619282 m6A levels. **(I, J)** The effect of mutated methylation sites on total m6A and ENST00000619282 m6A levels when FTO is overexpressed. **(K, L)** The influence of mutated methylation sites on cell proliferation and apoptosis when FTO is overexpressed. *p < 0.05, **p < 0.01, ***p < 0.001, ns, no significant.

The regulatory role of FTO in the proliferation and apoptosis of co-cultured RA-FLS cells was investigated. Gene editing experiments revealed that FTO expression levels notably influenced cell proliferation. Briefly, silencing FTO inhibited cell proliferation, while FTO overexpression promoted cell proliferation (**Figure 3A**). FCM further demonstrated that changes in FTO expression affected cell apoptosis, with FTO silencing increasing the apoptosis rate and its overexpression reducing cell apoptosis (**Figure 3B**). WB results showed that compared to the si-NC group, the si-FTO group exhibited increased Bax protein level and decreased Bcl-2 protein level, further confirming the role of FTO in promoting cell apoptosis escape; conversely, the OE-FTO group exhibited the opposite protein expression pattern (**Figure 3C**). Notably, FTO regulated the m6A modification level of ENST00000619282. Silencing FTO led to increased m6A modification and decreased expression of ENST00000619282, while overexpression of FTO exerted opposite effects (**Figures 3D, E**). This suggested that FTO might promote the expression of ENST00000619282 by downregulating its m6A modification.

To validate this finding, we successfully predicted two m6A methylation mutation sites on the ENST00000619282 sequence using the online database SRAMP (http://www.cuilab.cn/sramp/). Furthermore, mutation experiments were conducted. The results showed that m6A levels of ENST00000619282 were decreased upon mutation, with a more significant reduction observed when both sites were mutated simultaneously (**Figures 3G, H**). The role of m6A methylation in FTO-mediated regulation of cell fate was further determined. In short, we overexpressed FTO while mutating these two methylation sites. The experimental results indicated a significant reduction in both overall m6A and ENST00000619282 m6A levels (**Figures 3I, J**). Furthermore, cell viability was markedly decreased under these conditions (**Figure 3K**). FCM analysis results also revealed that the apoptosis rate in the OE-FTO + MUT group was significantly higher compared to that in the OE-FTO + WT group (**Figure 3L**). These results further confirmed the significant role of m6A methylation in the FTO-mediated promotion of RA-FLS proliferation and apoptosis escape.

### 3.4 FTO regulates ENST00000619282 through m6A modification, thereby mediating a YTHDF1-dependent mechanism to promote co-cultured RA-FLS proliferation and apoptosis escape

**Figure 4:**
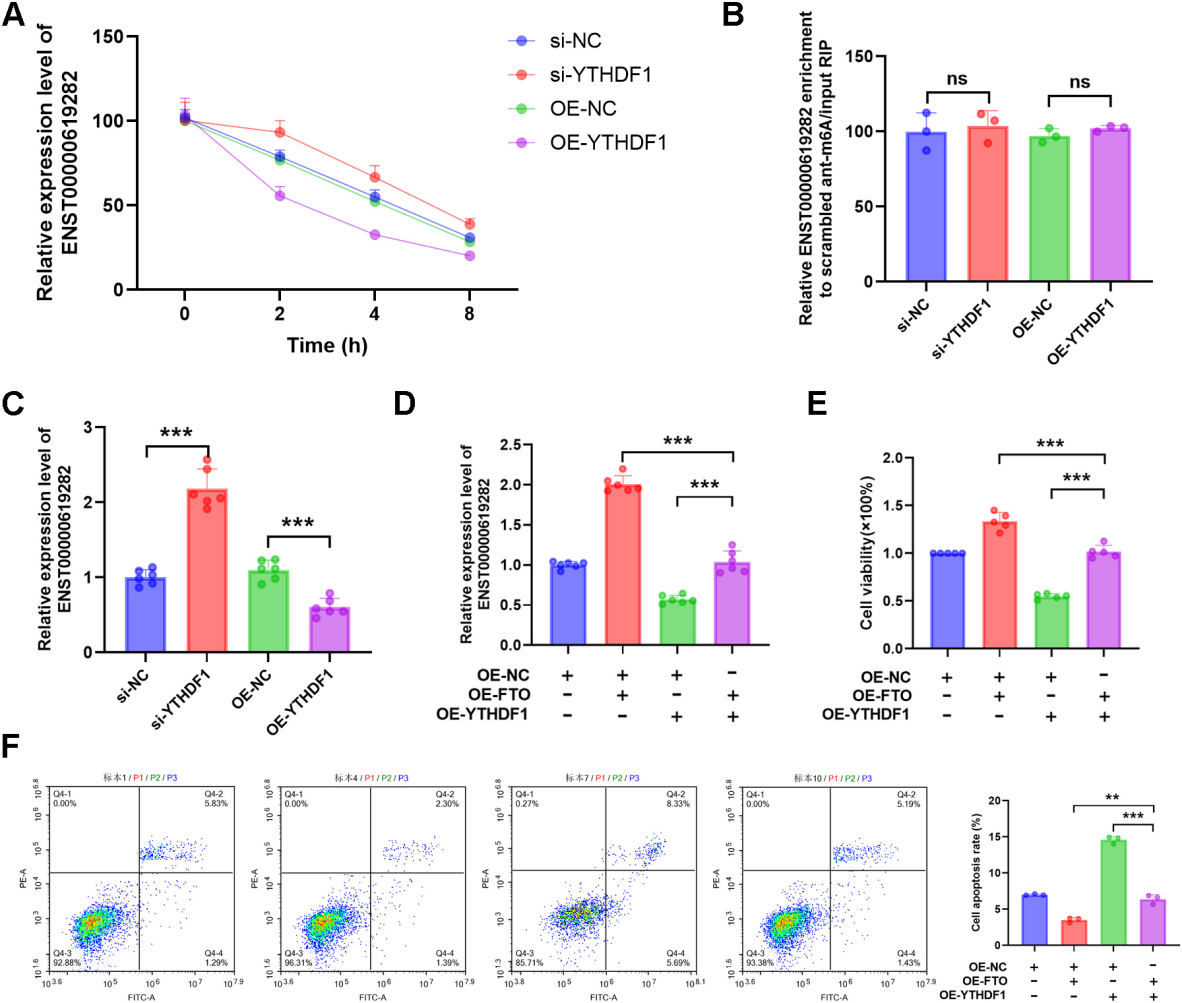
FTO regulates ENST00000619282 through m6A modification, mediating a YTHDF1-dependent mechanism to promote co-cultured RA-FLS proliferation and apoptosis escape. **(A)** The impact of YTHDF1 silencing or overexpression on the stability of ENST00000619282. **(B, C)** The influence of YTHDF1 knockdown/overexpression on the m6A modification and expression levels of ENST00000619282. **(D)** The effect of overexpressing YTHDF1 and FTO on ENST00000619282. **(E, F)** The impact of overexpressing YTHDF1 and FTO on cell proliferation and apoptosis. **p < 0.01, ***p < 0.001, ns, no significant.

To delve deeper into the role of YTHDF1 in regulating the stability of ENST00000619282, we conducted an actinomycin D experiment. As demonstrated by the results, when YTHDF1 was silenced, the half-life of ENST00000619282 mRNA was significantly extended, leading to increased stability. Conversely, overexpression of YTHDF1 shortened the half-life of this mRNA, thus reducing its stability (**Figure 4A**). Notably, the knockdown or overexpression of YTHDF1 had a negligible impact on the m6A modification level of ENST00000619282 (**Figure 4B**). RT-qPCR analysis results revealed that silencing YTHDF1 promoted the expression of ENST00000619282, while YTHDF1 overexpression inhibited ENST00000619282 expression (**Figure 4C**). These findings indicated that YTHDF1 primarily affected the RNA stability of ENST00000619282, rather than directly altering its m6A modification level.

Furthermore, when YTHDF1 was overexpressed alongside FTO, it was found that FTO significantly upregulated ENST00000619282 expression (**Figure 4D**), suggesting that FTO may indirectly enhance the stability of ENST00000619282 by downregulating YTHDF1 expression. Furthermore, cell proliferation and apoptosis experiments were conducted to clarify the role of YTHDF1 in FTO-mediated cellular functions. The results showed that overexpressing FTO in the context of YTHDF1 overexpression markedly reversed the inhibitory effect of YTHDF1 on cell proliferation (**Figure 4E**). Simultaneously, YTHDF1-promoted cell apoptosis was also significantly inhibited by FTO overexpression (**Figure 4F**). In summary, our study revealed a YTHDF1-dependent mechanism regulated by FTO through m6A modification, playing a pivotal role in promoting RA-FLS cell proliferation and apoptosis escape.

### 3.5 ENST00000619282 promotes co-cultured RA-FLS proliferation and apoptosis escape by inhibiting PUF60

**Figure 5:**
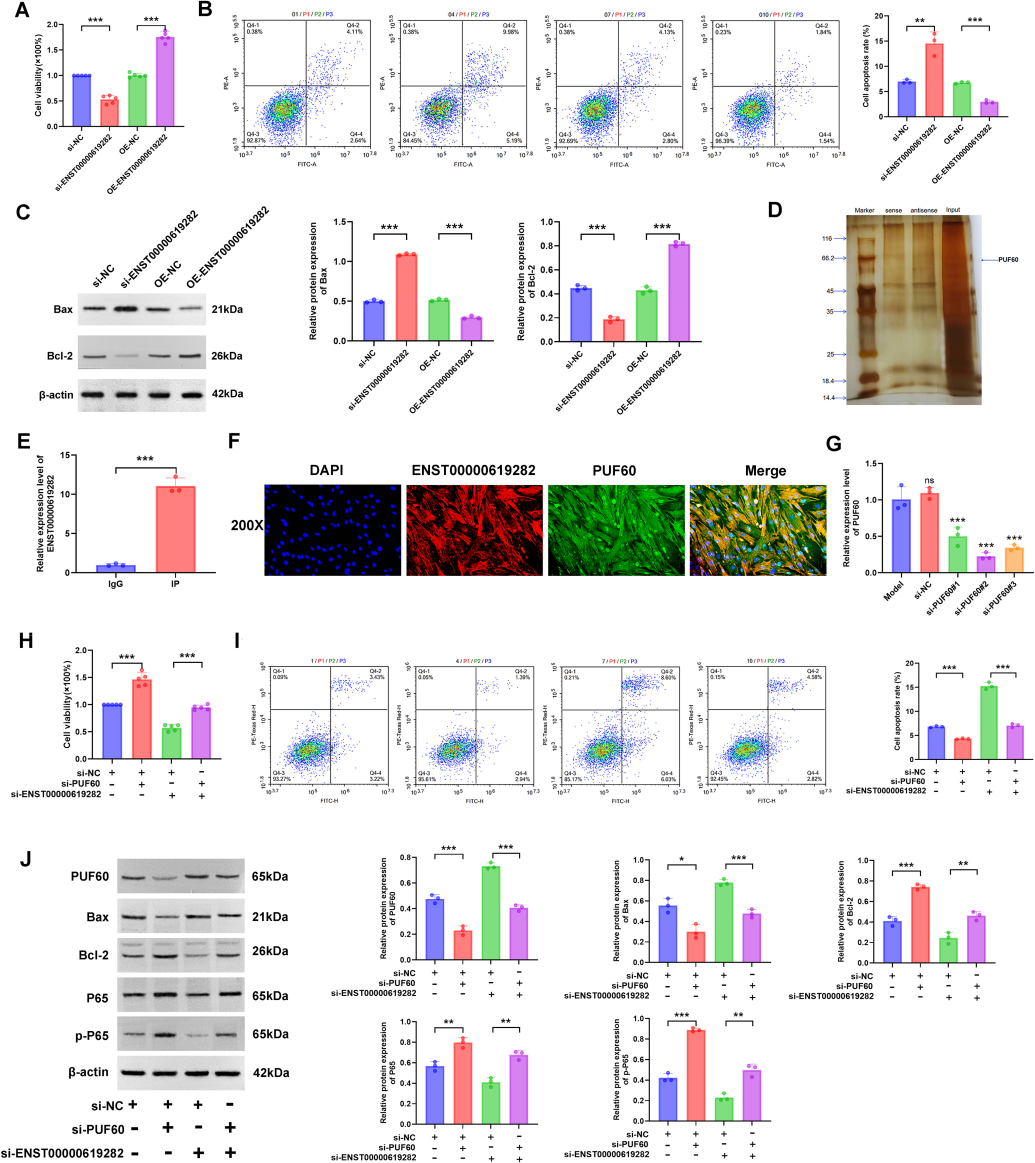
ENST00000619282 promotes the proliferation and apoptosis escape of co-cultured RA-FLS by inhibiting PUF60. **(A)** The impact of knockdown/overexpression of ENST00000619282 on the proliferation of RA-FLS cells. **(B)** The impact of knockdown/overexpression of ENST00000619282 on the apoptosis of RA-FLS cells. **(C)** The impact of knockdown/overexpression of ENST00000619282 on apoptosis-related proteins (Bax and Bcl-2). **(D)** RNA pull-down experiment identifying PUF60 as a binding protein of ENST00000619282. **(E)** RIP experiment demonstrating the interaction between ENST00000619282 and PUF60. **(F)** FISH experiment confirming the co-localization of ENST00000619282 and PUF60 (DAPI: nucleus, red light: ENST00000619282, green light: PUF60, Merge: combination of DAPI, ENST00000619282, and PUF60). **(G)** Screening of three silencing sequences for PUF60. **(H, I)** The impact of knocking down ENST00000619282 and PUF60 on cell proliferation and apoptosis. **(J)** The impact of knocking down ENST00000619282 and PUF60 on the expression level of PUF60, Bax, Bcl-2, P65, and p-P65 proteins. *p < 0.05, **p < 0.01, ***p < 0.001.

Subsequently, a series of detailed experiments were conducted for an in-depth exploration of the regulatory mechanisms of ENST00000619282 in promoting the proliferation and apoptosis escape of co-cultured RA-FLS cells. Firstly, the CCK-8 assay was performed, and it was found that the knockdown of ENST00000619282 significantly decreased cell viability, while ENST00000619282 overexpression notably enhanced cell viability (**Figure 5A**). This result directly confirmed the promoting role of ENST00000619282 in the proliferation of co-cultured RA-FLS cells. Next, FCM was employed to analyze cell apoptosis, and the results showed that silencing of ENST00000619282 significantly increased the apoptosis rate, while ENST00000619282 overexpression markedly decreased cell apoptosis (**Figure 5B**). This indicated that ENST00000619282 promoted apoptosis escape in co-cultured RA-FLS cells. Furthermore, changes in the expression of apoptosis-related proteins were examined. It was found that silencing ENST00000619282 led to an increase in Bax expression and a decrease in Bcl-2 expression, whereas ENST00000619282 overexpression reversed the expression of these proteins (**Figure 5C**), further supporting the above conclusions.

To reveal the molecular mechanism of the role of ENST00000619282, we utilized RNA pull-down technology to identify its binding protein PUF60 (**Figure 5D**). Moreover, the interaction and co-localization between ENST00000619282 and PUF60 were verified through RIP and FISH experiments, respectively (**Figures 5E, F**). This evidence collectively suggested that ENST00000619282 exerted its biological functions by binding to PUF60. Next, the most effective PUF60 silencing sequence was screened to further investigate whether ENST00000619282 regulated cell proliferation and apoptosis through PUF60 (**Figure 5G**). Under conditions of simultaneous silencing of ENST00000619282 and PUF60, we observed significantly enhanced cell proliferation (**Figure 5H**) and inhibited cell apoptosis (**Figure 5I**), accompanied by downregulated Bax and PUF60 expression levels and upregulated Bcl-2, P65, and p-P65 expression levels (**Figure 5J**). These findings not only confirmed that ENST00000619282 promoted RA-FLS cell proliferation and apoptosis escape by inhibiting PUF60 but also suggested that this process may be closely related to the activation of the NF-κB signaling pathway.

### 3.6 XFC inhibits co-cultured RA-FLS proliferation and apoptosis escape by regulating the FTO/ENST00000619282/NF-κB axis

**Figure 6:**
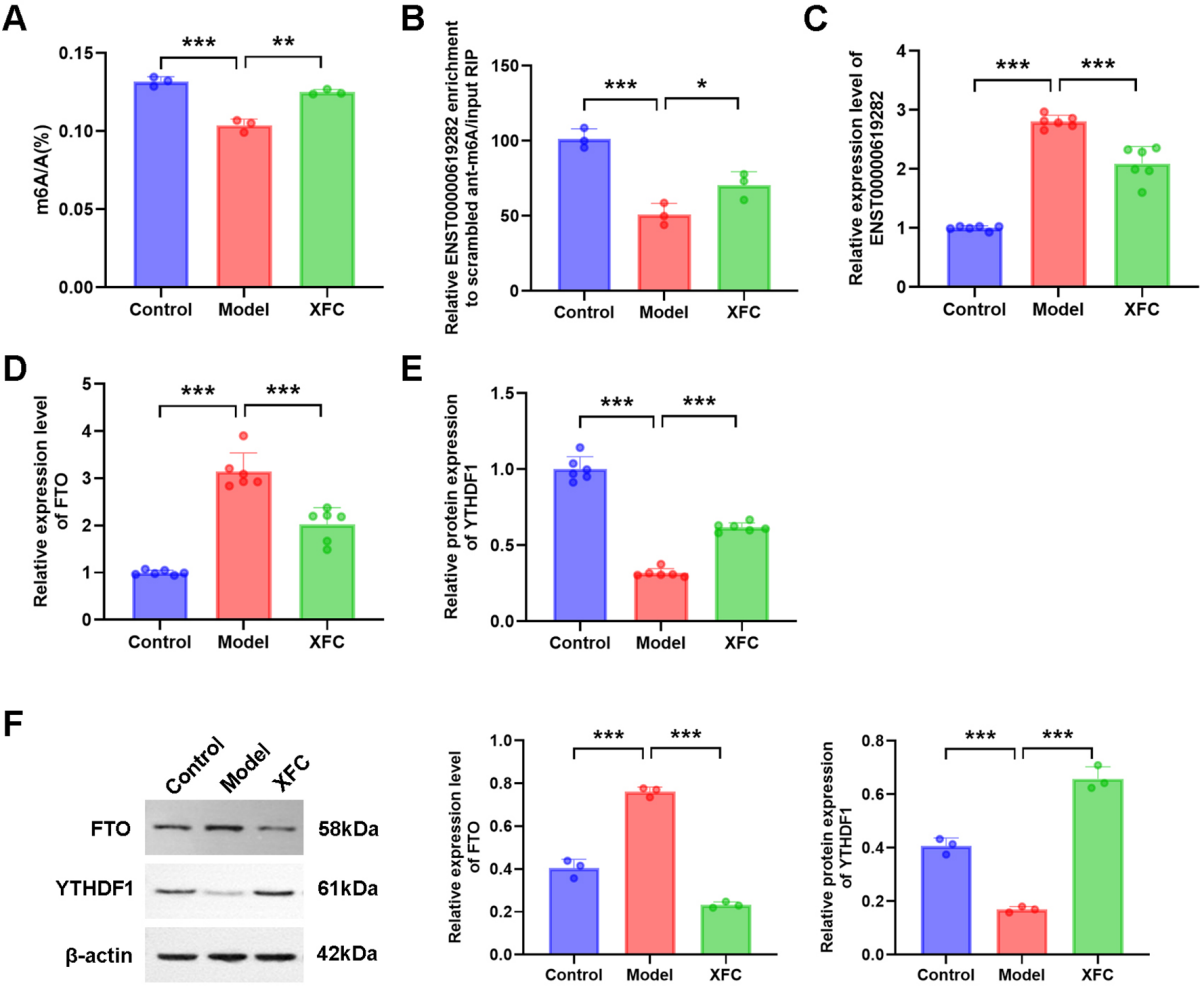
The function of XFC is analyzed. **(A–F)** The effect of XFC-containing serum on the expression of total m6A, ENST0000619282 m6A, ENST0000619282, FTO, and YTHDF1. *p < 0.05, **p < 0.01, ***p < 0.001.

In our interventional study, XFC-containing serum exhibited a series of regulatory effects on cell proliferation and apoptosis escape. Specifically, XFC-containing serum markedly increased the total m6A level, m6A modification level of ENST00000619282, and YTHDF1 expression (**Figures 6A, B, E**), while inhibiting ENST00000619282 and FTO expressions (**Figures 6C, D**). Furthermore, WB was conducted to further verify these gene-level changes at the protein level. It was found that XFC-containing serum indeed downregulated FTO protein level and upregulated YTHDF1 protein level (**Figure 6F**).

**Figure 7:**
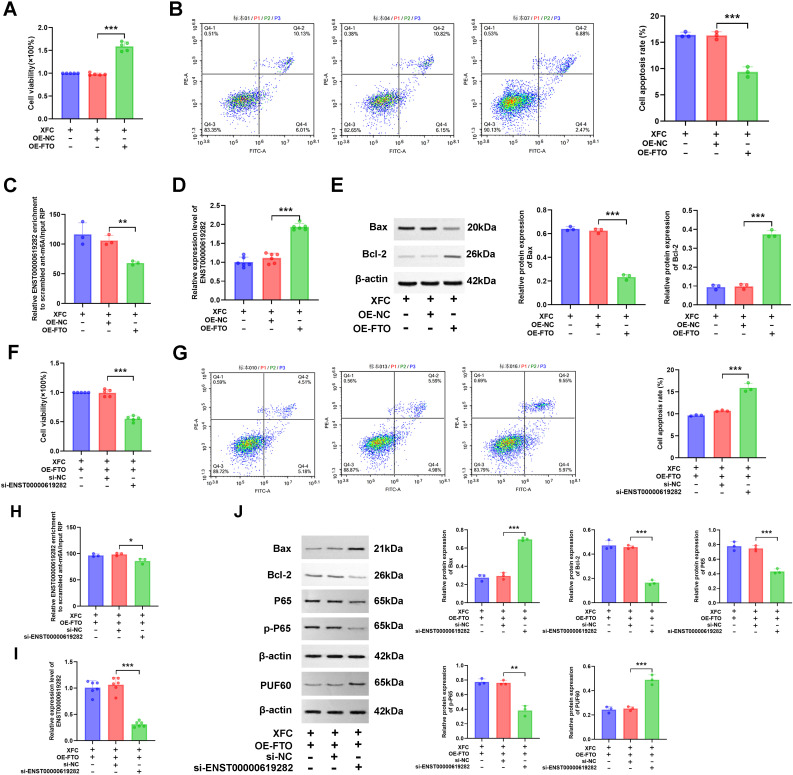
XFC inhibits co-cultured RA-FLS proliferation and apoptosis escape by regulating the FTO/ENST00000619282/NF-κB Axis. **(A)** Effects of overexpressing FTO on cell viability with XFC intervention. **(B)** Effects of overexpressing FTO on cell apoptosis with XFC intervention. **(C)** Effects of overexpressing FTO on the m6A modification of ENST00000619282 with XFC intervention. **(D)** Effects of overexpressing FTO on ENST00000619282 expression with XFC intervention. **(E)** Effects of overexpressing FTO on apoptotic proteins (Bax and Bcl-2) with XFC intervention. **(F)** Effects of overexpressing FTO and silencing ENST00000619282 on cell viability with XFC intervention. **(G)** Effects of overexpressing FTO and silencing ENST00000619282 on cell apoptosis with XFC intervention. **(H)** Effects of overexpressing FTO and silencing ENST00000619282 on the m6A modification of ENST00000619282 with XFC intervention. **(I)** Effects of overexpressing FTO and silencing ENST00000619282 on ENST00000619282 expression with XFC intervention. **(J)** Effects of overexpressing FTO and silencing ENST00000619282 on apoptotic proteins (Bax and Bcl-2), p65, p-p65, and PUF60 with XFC intervention. *p < 0.05, **p < 0.01, ***p < 0.001.

Subsequently, a series of rescue experiments were performed to delve deeper into the underlying mechanism. Firstly, based on XFC intervention, an FTO overexpression experiment was conducted. The results showed that FTO overexpression increased cell viability (**Figure 7A**) and decreased cell apoptosis rate (**Figure 7B**), accompanied by reduced m6A modification level of ENST00000619282 (**Figure 7C**) and increased expression of ENST00000619282 (**Figure 7D**). Additionally, FTO overexpression reduced Bax protein level and increased Bcl-2 protein level (**Figure 7E**), both indicating the inhibition of cell apoptosis. To further explore the role of ENST00000619282 in this process, we knocked down ENST00000619282 based on XFC intervention and FTO overexpression. This combined intervention effectively reversed the effects of FTO overexpression alone on cell viability, cell apoptosis rate, m6A modification of ENST00000619282, and ENST00000619282 expression level (**Figures 7F–I**). Based on this, the level of proteins such as PUF60, Bax, Bcl-2, P65, and p-P65 was detected through WB, and the results showed upregulated Bax and PUF60 protein levels, and downregulated Bcl-2, P65, and p-P65 levels (**Figure 7J**). In summary, our study suggested that XFC-containing serum may inhibit cell proliferation and apoptosis escape by suppressing the FTO/ENST00000619282/NF-κB axis.

### 3.7 The expression levels of FTO/YTHDF1/ENST00000619282/Bax/Bcl-2 in RA patients are closely associated with immune-inflammatory indicators

**Figure 8:**
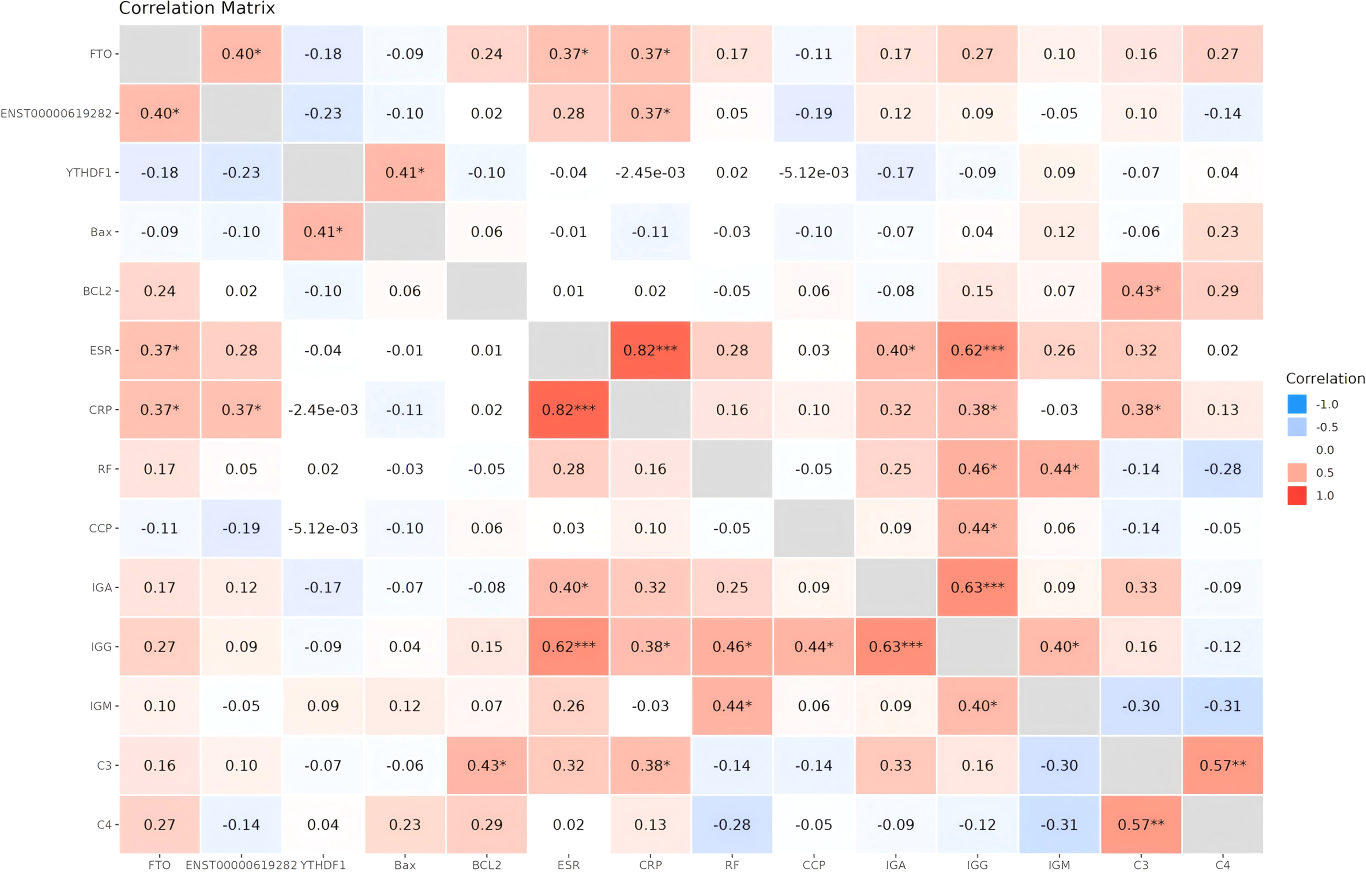
Heatmap of correlation analysis between FTO/YTHDF1/ENST00000619282/Bax/Bcl-2 and immune-inflammatory indicators. *P<0.05, **P<0.01, ***P<0.001.

The role of FTO/YTHDF1/ENST00000619282/Bax/Bcl-2 in RA was further validated. Briefly, blood samples from 30 RA patients were collected and assessed for the expression levels of FTO/YTHDF1/ENST00000619282/Bax/Bcl-2. Furthermore, the correlation between FTO/YTHDF1/ENST00000619282/Bax/Bcl-2 and immune-inflammatory indicators in RA patients was analyzed (**Figure 8**). The results revealed significant positive correlations between FTO and both ENST00000619282 and CRP; between YTHDF1 and Bax; between ENST00000619282 and CRP; and between Bcl-2 and C3. Additionally, RF was positively correlated with IgG, and IgM; IgG correlated with IgM; IgA correlated with IgG; ESR correlated with IgA, CRP, and IgG; CRP correlated with IgG and C3; CCP correlated with IgG; and C3 correlated with C4 (*p* < 0.05). Specific rho values and p-values are provided in **Supplementary Table 1**. These findings suggested that FTO/YTHDF1/ENST00000619282/Bax/Bcl-2 were closely associated with the immune-inflammatory status of RA patients, and there may also be strong correlations among the immune-inflammatory indicators.

### 3.8 XFC improves the levels of FTO/YTHDF1/ENST00000619282/Bax/Bcl-2 and immune-inflammatory indicators

It was observed that XFC treatment notably decreased FTO, ENST00000619282, Bcl-2, ESR, CRP, and C4 expression levels compared to pre-treatment levels. Conversely, the expressions of YTHDF1 and Bax were significantly increased after XFC treatment (**Table 3**). The results of clinical validation were consistent with the expression trends observed in cellular experiments. However, there were no statistically significant differences in RF, CCP, IgA, IgG, and IgM levels. Additionally, the impact of XFC on ALT and AST levels was assessed and no significant changes were observed before and after treatment. These findings suggested that XFC treatment exerted a significant impact on the levels of FTO, YTHDF1, ENST00000619282, Bax, and Bcl-2, as well as some immune-inflammatory indicators.

**Table 3:** Changes in FTO/YTHDF1/ENST00000619282/Bax/Bcl-2 and immune-inflammatory indicators before and after treatment (n = 30).

Indexes	Control (n=30)	XFC (n=30)	*p*-value
FTO	0.69 (0.60, 0.85)	0.59 (0.50, 0.65)	0.003
ENST00000619282	0.75 ± 0.25	0.49 ± 0.24	<0.001
YTHDF1	0.29 (0.23, 0.49)	0.66 (0.49, 0.87)	<0.001
Bax	0.55 (0.43, 0.73)	0.88 (0.58, 1.02)	0.002
BCL2	2.00 ± 0.59	1.40 ± 0.56	<0.001
ESR	43 (27, 72)	33 (20, 45)	0.050
CRP	13 (8, 35)	8 (3, 20)	0.033
RF	66 (24, 165)	57 (22, 174)	0.663
CCP	75 (17, 180)	61 (17, 169)	0.630
IGA	2.92 (2.35, 3.55)	2.71 (2.11, 3.47)	0.455
IGG	11.0 (8.5, 13.8)	10.7 (8.3, 13.6)	0.784
IGM	1.13 (0.89, 1.60)	1.15 (0.86, 1.42)	0.853
C3	1.18 ± 0.19	1.10 ± 0.18	0.108
C4	0.33 (0.27, 0.37)	0.27 (0.20, 0.33)	0.024
ALT	11 (8, 16)	14 (11, 18)	0.063
AST	15 (13, 19)	17 (14, 26)	0.101

### 3.9 Association rule analysis is conducted between XFC treatment and improvements in FTO/YTHDF1/ENST00000619282/Bax/Bcl-2 and immune-inflammatory indicators

All 30 RA patients received XFC treatment. With a minimum confidence level set at 40%, the association rule results revealed that XFC treatment was significantly associated with increased levels of Bax and YTHDF1, with a confidence level exceeding 70% and a lift of 1. Additionally, XFC treatment was markedly associated with decreased levels of Bcl-2, FTO, ENST00000619282, RF, ESR, C4, C3, IgG, IgA, CRP, CCP, and IgM, with a confidence level exceeding 40% and a lift of 1 (**Table 4**). These results further demonstrated a strong correlation between XFC treatment and improvements in FTO/YTHDF1/ENST00000619282/Bax/Bcl-2 and immune-inflammatory indicators.

**Table 4:** Association rule analysis is conducted between XFC treatment and improvements in FTO/YTHDF1/ENST00000619282/Bax/Bcl-2 and immune-inflammatory indicators (n=30).

The Former	The consequent	Support(%)	Confidence(%)	Lift
XFC	Bax↑	100	80	1
XFC	Bcl-2↓	100	76.667	1
XFC	YTHDF1↑	100	73.333	1
XFC	FTO↓	100	70	1
XFC	ENST00000619282↓	100	70	1
XFC	RF↓	100	66.667	1
XFC	ESR↓	100	66.667	1
XFC	C4↓	100	63.333	1
XFC	C3↓	100	60	1
XFC	IGG↓	100	53.333	1
XFC	IGA↓	100	53.333	1
XFC	CRP↓	100	46.667	1
XFC	CCP↓	100	43.333	1
XFC	IGM↓	100	43.333	1

The symbol '↑' indicates an increase in the indicator, while '↓' represents a decrease.

### 3.10 Molecular docking of active components in XFC with the key protein p65 in the NF-κB signaling pathway is conducted

**Figure 9:**
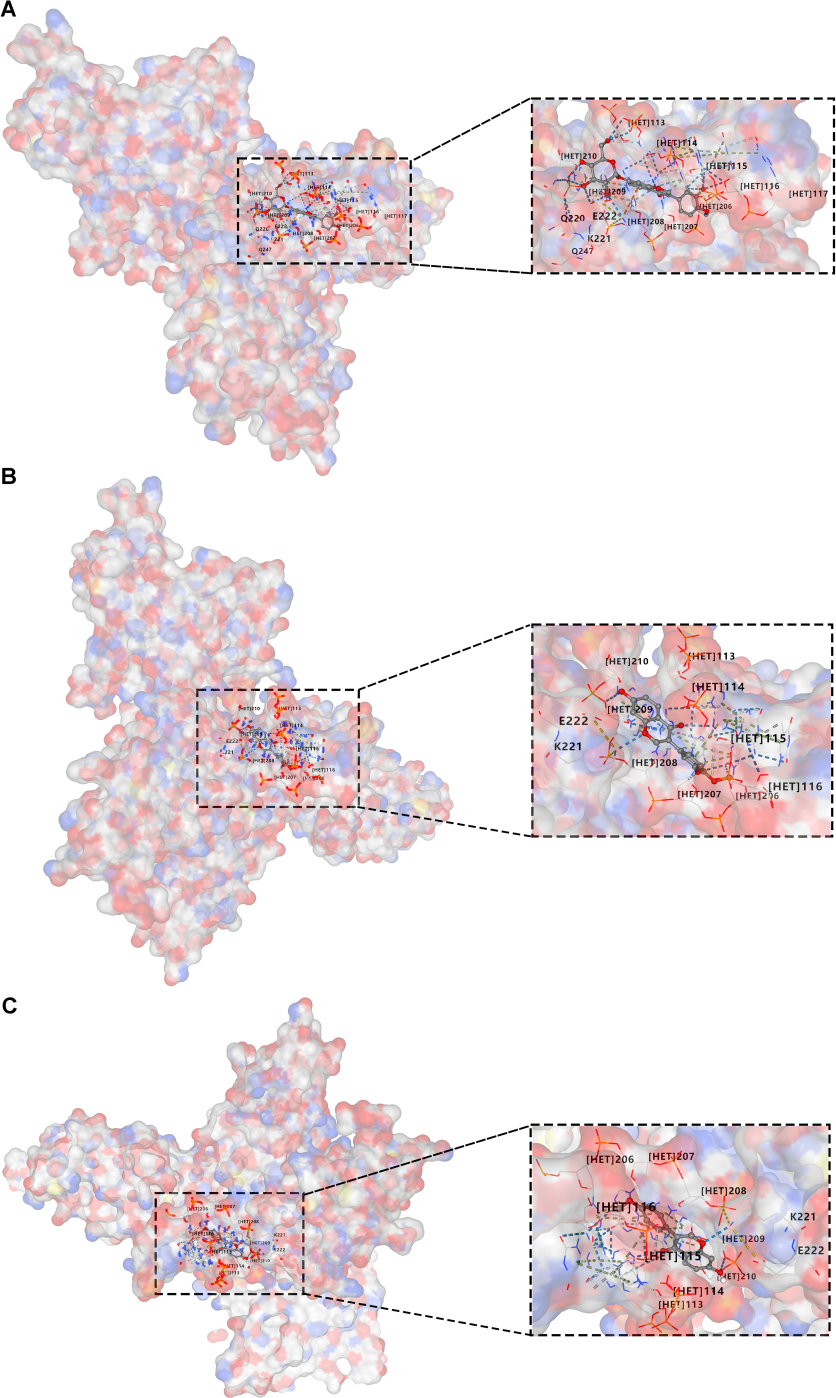
Molecular docking of active components in XFC with the key protein p65 in the NF-κB signaling pathway is conducted. **(A)** Docking of calycosin-7-O-beta-D-glucoside with P65. **(B)** Docking of calycosin with P65. **(C)** Docking of formononetin with P65.

Our previous research has analyzed the main components of XFC using HPLC fingerprint technology and identified three hallmark active components, including calycosin-7-O-beta-D-glucoside, calycosin, and formononetin (22). Moreover, molecular docking was further conducted using CB-Dock2 software to further investigate the binding interactions between XFC and the key protein P65 in the NF-κB signaling pathway (**Figure 9**). The experimental results demonstrated that these three active components (calycosin-7-O-beta-D-glucoside, calycosin, and formononetin) exhibited good binding affinities with P65. Specifically, calycosin-7-O-beta-D-glucoside tightly bound to the active pocket of P65 (key residues: ARG33, ASN186, ARG187, etc.), with a minimum binding energy of -10.8 kcal/mol. Calycosin also showed tight binding to the active pocket of P65 (key residues: ASN186, ARG187, LYS218, etc.), with a minimum binding energy of -9.0 kcal/mol. Similarly, formononetin bound tightly to the active pocket of P65 (key residues: GLN220, LYS221, GLU222, etc.), with a minimum binding energy of -8.2 kcal/mol (**Supplementary Figure 1**).

## 4 Discussion

RA is a complex autoimmune disease characterized by abnormal proliferation and inflammation of synovial tissue (31). In normal conditions, synovial tissue lubricates joints and cartilage, providing them with necessary nourishment. However, excessive proliferation of synovial tissue becomes the primary cause of joint destruction in RA patients (32). This process involves synovial cell proliferation, immune cell infiltration, and their interactions, leading to synovial intimal hyperplasia, intimal cell proliferation, interstitial inflammatory cell infiltration, and pannus formation, ultimately damaging cartilage and bone tissue (33). Studies have shown that FLS in synovial tissue are the main effector cells causing cartilage damage and destruction, exhibiting tumorigenic-like invasiveness; escape from apoptosis is an important inducer of RA pathogenesis (5, 34). Additionally, it has been reported that m6A RNA methylation may be tightly associated with RA and play a crucial role in regulating various biological processes of human diseases, such as cell activation, proliferation, and apoptosis (35). Therefore, in this study, *in-vivo* experiments and clinical validations were conducted to deeply explore the importance of m6A RNA methylation in RA and elucidate its specific mechanisms in RA-FLS cell proliferation and apoptosis escape.

m6A modification is not only a crucial regulatory mechanism in RNA internal metabolism, involving mRNA stability, splicing, translation, and other aspects but is closely related to the pathogenesis of various diseases, including cancer and autoimmune diseases (36, 37). Research has found that demethylase FTO can affect cell apoptosis in different disease models. For example, *in-vitro* cell experiments in previous studies have demonstrated that FTO inhibits apoptosis in osteosarcoma cells and myoblasts (38, 39). Moreover, similar results have also been verified in bladder cancer (40). Furthermore, increased FTO expression in RA synovial cells promotes their proliferation and migration and reduces senescence and apoptosis, while inhibition of FTO significantly slows disease progression (41). Similar results were also observed in this study. Specifically, in a coculture model of RA-PBMCs and RA-FLSs, we found that FTO expression was significantly increased and mediated a decrease in the m6A modification level of ENST00000619282, thereby upregulating ENST00000619282 expression. This suggested that m6A modification may play a complex regulatory role in this model, and the increase in FTO expression levels may be the primary cause of this modification change. Additionally, as an m6A reader protein, YTHDF1 downregulation may further affect the degradation or translation regulation of m6A-modified RNA, indirectly promoting ENST00000619282 expression.

Gene editing experiments in this study further confirmed the key role of FTO in regulating the proliferation and apoptosis escape of co-cultured RA-FLS cells. Silencing of FTO significantly inhibited cell proliferation and promoted cell apoptosis, while overexpression of FTO exhibited the opposite effects. This regulatory process was closely related to the m6A modification level of ENST00000619282. Simultaneously, it was also found that FTO may affect the expression of ENST00000619282 by regulating m6A methylation sites, thereby participating in the regulation of cell proliferation and apoptosis. On the other hand, YTHDF1, as an m6A reader protein, has been shown to play an important role in regulating RNA stability and degradation (42). This study found that silencing YTHDF1 markedly prolonged the half-life of ENST00000619282 mRNA, increasing its stability, while overexpression of YTHDF1 caused the opposite effect. Although interference or overexpression of YTHDF1 exerted no significant effect on the m6A modification level of ENST00000619282, its regulation of RNA stability directly affected the expression level of this gene. This phenomenon may be related to the functional characteristics of YTHDF1 and its role in the dynamic balance of m6A modification. YTHDF1 affects the degradation rate of RNA by binding to m6A sites rather than directly participating in the formation or removal of m6A modifications. Furthermore, RNA stability may also be regulated by other factors (such as RNA-binding proteins or RNA secondary structures), which may represent another potential mechanism of YTHDF1 in regulating ENST00000619282 expression. Further research indicated that FTO may indirectly enhance the stability of ENST00000619282 by downregulating YTHDF1 expression, thereby participating in the regulatory process of cell proliferation and apoptosis.

Through RNA sequencing and bioinformatics analysis in our preliminary research, the apoptosis-related gene ENST00000619282 was identified. Further studies have revealed that overexpression of ENST00000619282 elevates levels of pro-apoptotic and pro-inflammatory factors while decreasing levels of anti-apoptotic proteins and anti-inflammatory factors in RA-FLS (10, 19). This study found that silencing ENST00000619282 notably reduced the viability of co-cultured RA-FLS and promoted cell apoptosis, while ENST00000619282 overexpression caused the opposite effect. Through RNA pull-down technology, PUF60 was identified as a binding protein of ENST00000619282, and their interaction and co-localization were verified through RIP and FISH experiments. Furthermore, it was found that ENST00000619282 promoted the proliferation and apoptosis evasion of co-cultured RA-FLS by inhibiting PUF60-activated NF-κB signaling. This discovery not only reveals the critical role of ENST00000619282 in RA pathogenesis but also provides a theoretical basis for developing novel therapeutic strategies targeting this pathway.

XFC is a hospital preparation of the Anhui Provincial Hospital of Traditional Chinese Medicine, exhibiting various effects such as anti-inflammatory, antioxidant, and immunoregulatory properties, as well as promoting RA-FLS cell apoptosis and improving hypercoagulability (43–45). A previous study has characterized the fingerprint of XFC in detail using HPLC, clearly identifying calycosin-7-glucoside, calycosin, and formononetinaldehyde as its main active components (22). A prior study has confirmed that XFC is comparable to leflunomide in reducing multiple RA-related indicators and significantly outperforms leflunomide in improving the quality of life of RA patients (23). In this study, retrospective data mining techniques (including association rule analysis and random walk evaluations) were employed to assess the efficacy of XFC on clinical immune-inflammatory indicators in RA patients. The results indicated that the application of XFC was closely associated with the improvements in immune-inflammatory indicators in RA patients, showing significant long-range correlations. Further research revealed that XFC significantly inhibited the expression of FTO and ENST00000619282 while upregulating the expression of YTHDF1, thereby restoring m6A modification levels and affecting the expression of related genes. This discovery suggested that XFC may inhibit cell proliferation and apoptosis evasion by suppressing the FTO/ENST00000619282/NF-κB axis. This finding not only provides a theoretical basis for the application of XFC in RA treatment but also offers novel insights into developing therapeutic strategies based on m6A modification.

Moreover, this study further analyzed 30 clinical samples and validated the association between key molecules (such as FTO/YTHDF1/ENST00000619282/Bax/Bcl-2) and the immune-inflammatory status of RA patients. According to the results, these molecules were closely related to multiple immune-inflammatory indicators in RA patients, and XFC treatment remarkably improved the expression levels of these molecules and some immune-inflammatory indicators. Association rule analysis further confirmed the strong correlation between XFC treatment and the improvement of these molecules and immune-inflammatory indicators. These findings not only provide a new perspective for understanding RA pathogenesis but also provide robust support for the clinical application of XFC in RA treatment. Finally, this study explored the binding of the main active components of XFC to P65 (a key protein in the NF-κB signaling pathway) through molecular docking experiments. As revealed by the results, three main active components of XFC (calycosin-7-glucoside, calycosin, and formononetinaldehyde) exhibited good binding capacity with P65, suggesting that XFC may affect the activity of the NF-κB signaling pathway by directly acting on P65. This discovery provides new clues to the potential mechanism of XFC in RA treatment and lays a theoretical foundation for the further development of novel therapeutic strategies based on XFC.

**Figure 10:**
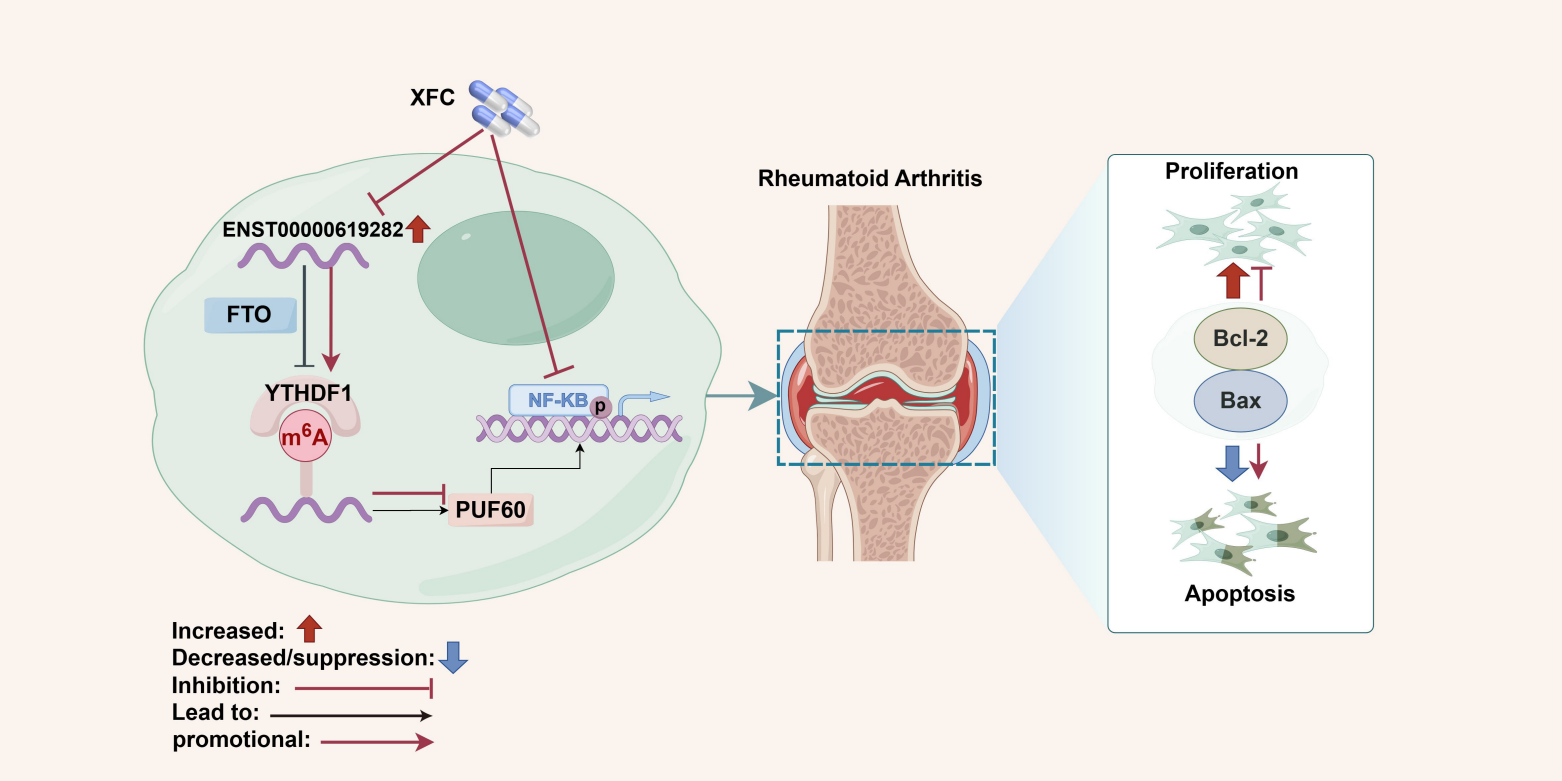
Schematic diagram summarizing the main findings of the present study. Created by Figdraw (ID: AOSRRf5e8f).

In summary, this study unveiled the pivotal roles of key molecules (such as FTO/YTHDF1/ENST00000619282/Bax/Bcl-2) in the pathogenesis of RA, as well as the potential mechanism of XFC in inhibiting cell proliferation and apoptosis evasion by regulating these molecules and the NF-κB signaling pathway through a series of experiments (**Figure 10**). The strengths of this study are mainly reflected in the following aspects. First, this study boasts an innovative research perspective. Specifically, this study explores the pathogenesis of RA and the therapeutic effect of XFC from a novel perspective by examining the role of lncRNA m6A modification, providing fresh insights and targets for RA research and treatment. Second, this study adopts a comprehensive research methodology. Various approaches (such as data mining, cellular experiments, bioinformatics predictions, clinical validation, and molecular docking) were integrated to establish a complete and rigorous research system, enhancing the accuracy and reliability of the study. Third, this study possesses a solid clinical efficacy foundation. The research team conducted a large-scale retrospective data mining analysis on 1603 patients and validated the expression of FTO/YTHDF1/ENST00000619282/Bax/Bcl-2 in clinical cohorts, further strengthening the clinical significance and application value of the research findings. Fourth, this study is distinctive in its cellular experimental design. The research team co-cultured PBMCs derived from clinical RA patients with RA-FLS and co-edited related genes, providing robust experimental support for in-depth exploration of the pathogenesis of RA and the therapeutic effect of XFC. Fifth, this study utilized molecular docking technology to verify the binding capacity of the active ingredients of XFC with the NF-κB pathway protein p65, providing direct molecular-level evidence for the mechanism of XFC in treating RA. However, there are also some limitations in the present study. First, there are certain limitations in sample selection. Although the study employed large-scale retrospective data mining analysis, the samples were sourced from only one hospital and only immune inflammation indicators and apoptosis indicators of RA patients were observed, which may limit the universality and applicability of the research findings. Second, the pathogenesis of RA and the therapeutic effect of XFC may involve multiple complex signaling pathways and molecular networks. Although this study has revealed one important pathway (the NF-κB signaling pathway), there may still be other unknown mechanisms that require further exploration. Additionally, XFC is a multi-component traditional Chinese medicine formula, and its efficacy may stem from the independent or synergistic effects of its components. The current study only conducted molecular docking for three active components without covering other potential active components in XFC (such as astragaloside from Astragalus membranaceus and coixenolide from Coicis Semen). This may overlook the potential regulatory effects of other components on the FTO/ENST00000619282 axis. Therefore, the regulatory effects of other components in XFC (such as extracts of Astragalus membranaceus and Coicis Semen) on the FTO/ENST00000619282 axis should be assessed in future studies and the scope of molecular docking should be expanded to cover more potential active components. Finally, there are also limitations in the experimental model. The experiments in this study were mainly conducted at the cellular level. Although the method of co-culturing RA-FLS cells was adopted, it may not fully simulate the complex environment in humans. Hence, the experimental results need further validation and in-depth study in animal models. In response to these limitations, animal experiments and large-sample, multi-center, randomized controlled clinical studies would be conducted in our future research, continuing to deepen and expand our efforts to achieve more comprehensive and in-depth research results.

## 5 Conclusion

In summary, we prove that FTO promotes the expression of ENST00000619282 by downregulating its m6A level, thereby activating the NF-κB pathway, promoting cell proliferation and apoptosis evasion, and participating in the pathogenesis of RA. On the other hand, XFC exerts its therapeutic effects by regulating FTO to upregulate the m6A level of ENST00000619282, thereby inhibiting the NF-κB pathway activation, suppressing apoptosis evasion in co-cultured RA-FLS, and inhibiting immune-inflammatory responses. These findings may provide reliable research data for the clinical application of XFC in the treatment of RA.

## Data Availability

The raw data supporting the conclusions of this article will be made available by the authors, without undue reservation.
