# Supplementary material for: Demethylase FTO mediates m6A modification of ENST00000619282 to promote apoptosis escape in rheumatoid arthritis and the intervention effect of Xinfeng Capsule
Source: Front Immunol. 2025 Mar 13;16:1556764. doi: 10.3389/fimmu.2025.1556764 (PMC11966437; doi:10.3389/fimmu.2025.1556764)
Supplement: Supplementary file 1 [file Table1.docx]

Supplementary Table 1: Correlation analysis results (n=30)

| Parameter1 | Parameter2 | rho | CI | CI_low | CI_high | S | *P*-value |
| --- | --- | --- | --- | --- | --- | --- | --- |
| FTO | ENST00000619282 | 0.395773081 | 0.95 | 0.03027142542 | 0.66791716 | 2,716.0000 | 0.030 |
| FTO | YTHDF1 | -0.179532814 | 0.95 | -0.51524629858 | 0.20394600 | 5,302.0000 | 0.342 |
| FTO | Bax | -0.090100111 | 0.95 | -0.44519497684 | 0.28948233 | 4,900.0000 | 0.636 |
| FTO | BCL2 | 0.244048943 | 0.95 | -0.13837775604 | 0.56314116 | 3,398.0000 | 0.194 |
| FTO | ESR | 0.366751996 | 0.95 | -0.00368112632 | 0.64867712 | 2,846.4498 | 0.046 |
| FTO | CRP | 0.369966630 | 0.95 | 0.00003816381 | 0.65082621 | 2,832.0000 | 0.044 |
| FTO | RF | 0.171078977 | 0.95 | -0.21229010222 | 0.50881109 | 3,726.0000 | 0.366 |
| FTO | CCP | -0.107103138 | 0.95 | -0.45885644825 | 0.27367380 | 4,976.4286 | 0.573 |
| FTO | IGA | 0.171542998 | 0.95 | -0.21183354928 | 0.50916529 | 3,723.9142 | 0.365 |
| FTO | IGG | 0.273859844 | 0.95 | -0.10690431946 | 0.58457069 | 3,264.0000 | 0.143 |
| FTO | IGM | 0.098553949 | 0.95 | -0.28164818981 | 0.45200813 | 4,052.0000 | 0.604 |
| FTO | C3 | 0.164124345 | 0.95 | -0.21911280548 | 0.50348866 | 3,757.2611 | 0.386 |
| FTO | C4 | 0.268048454 | 0.95 | -0.11310027528 | 0.58042692 | 3,290.1222 | 0.152 |
| ENST00000619282 | YTHDF1 | -0.227141268 | 0.95 | -0.55079333166 | 0.15589155 | 5,516.0000 | 0.227 |
| ENST00000619282 | Bax | -0.103003337 | 0.95 | -0.45557751611 | 0.27750450 | 4,958.0000 | 0.588 |
| ENST00000619282 | BCL2 | 0.023804227 | 0.95 | -0.34920455230 | 0.39030093 | 4,388.0000 | 0.901 |
| ENST00000619282 | ESR | 0.284855919 | 0.95 | -0.09509910467 | 0.59236725 | 3,214.5726 | 0.127 |
| ENST00000619282 | CRP | 0.370856507 | 0.95 | 0.00106955263 | 0.65142033 | 2,828.0000 | 0.044 |
| ENST00000619282 | RF | 0.045161290 | 0.95 | -0.33029045654 | 0.40827407 | 4,292.0000 | 0.813 |
| ENST00000619282 | CCP | -0.185927484 | 0.95 | -0.52008901383 | 0.19759710 | 5,330.7440 | 0.325 |
| ENST00000619282 | IGA | 0.121704306 | 0.95 | -0.25993218577 | 0.47045684 | 3,947.9391 | 0.522 |
| ENST00000619282 | IGG | 0.094549499 | 0.95 | -0.28536542904 | 0.44878598 | 4,070.0000 | 0.619 |
| ENST00000619282 | IGM | -0.051390434 | 0.95 | -0.41346374180 | 0.32471646 | 4,726.0000 | 0.787 |
| ENST00000619282 | C3 | 0.096425836 | 0.95 | -0.28362509029 | 0.45029691 | 4,061.5659 | 0.612 |
| ENST00000619282 | C4 | -0.144385202 | 0.95 | -0.48824052438 | 0.23827548 | 5,144.0115 | 0.447 |
| YTHDF1 | Bax | 0.412235818 | 0.95 | 0.04991401111 | 0.67867207 | 2,642.0000 | 0.024 |
| YTHDF1 | BCL2 | -0.103003337 | 0.95 | -0.45557751611 | 0.27750450 | 4,958.0000 | 0.588 |
| YTHDF1 | ESR | -0.041615669 | 0.95 | -0.40530960163 | 0.33345152 | 4,682.0624 | 0.827 |
| YTHDF1 | CRP | -0.002447164 | 0.95 | -0.37204404542 | 0.36781951 | 4,506.0000 | 0.990 |
| YTHDF1 | RF | 0.023359288 | 0.95 | -0.34959539018 | 0.38992350 | 4,390.0000 | 0.902 |
| YTHDF1 | CCP | -0.005121356 | 0.95 | -0.37434582345 | 0.36550480 | 4,518.0205 | 0.979 |
| YTHDF1 | IGA | -0.167983091 | 0.95 | -0.50644497085 | 0.21533188 | 5,250.0840 | 0.375 |
| YTHDF1 | IGG | -0.086095662 | 0.95 | -0.44195327781 | 0.29317558 | 4,882.0000 | 0.651 |
| YTHDF1 | IGM | 0.090545050 | 0.95 | -0.28907127330 | 0.44555459 | 4,088.0000 | 0.634 |
| YTHDF1 | C3 | -0.065916969 | 0.95 | -0.42547547770 | 0.31161543 | 4,791.2968 | 0.729 |
| YTHDF1 | C4 | 0.041221084 | 0.95 | -0.33380278627 | 0.40497922 | 4,309.7112 | 0.829 |
| Bax | BCL2 | 0.057619577 | 0.95 | -0.31911620860 | 0.41862999 | 4,236.0000 | 0.762 |
| Bax | ESR | -0.012462446 | 0.95 | -0.38064127306 | 0.35912692 | 4,551.0187 | 0.948 |
| Bax | CRP | -0.113236930 | 0.95 | -0.46374429829 | 0.26791997 | 5,004.0000 | 0.551 |
| Bax | RF | -0.030033370 | 0.95 | -0.39557211261 | 0.34371916 | 4,630.0000 | 0.875 |
| Bax | CCP | -0.096415091 | 0.95 | -0.45028826250 | 0.28363506 | 4,928.3858 | 0.612 |
| Bax | IGA | -0.072088108 | 0.95 | -0.43054022793 | 0.30600610 | 4,819.0360 | 0.705 |
| Bax | IGG | 0.044271413 | 0.95 | -0.33108461022 | 0.40753077 | 4,296.0000 | 0.816 |
| Bax | IGM | 0.121245829 | 0.95 | -0.26036603644 | 0.47009441 | 3,950.0000 | 0.523 |
| Bax | C3 | -0.059013503 | 0.95 | -0.41978287682 | 0.31785940 | 4,760.2657 | 0.757 |
| Bax | C4 | 0.225713287 | 0.95 | -0.15735977804 | 0.54974394 | 3,480.4188 | 0.230 |
| BCL2 | ESR | 0.012017359 | 0.95 | -0.35951459736 | 0.38026055 | 4,440.9820 | 0.950 |
| BCL2 | CRP | 0.023804227 | 0.95 | -0.34920455230 | 0.39030093 | 4,388.0000 | 0.901 |
| BCL2 | RF | -0.051835373 | 0.95 | -0.41383353437 | 0.32431731 | 4,728.0000 | 0.786 |
| BCL2 | CCP | 0.063905614 | 0.95 | -0.31343801249 | 0.42381985 | 4,207.7443 | 0.737 |
| BCL2 | IGA | -0.078985427 | 0.95 | -0.43617437547 | 0.29970547 | 4,850.0395 | 0.678 |
| BCL2 | IGG | 0.152391546 | 0.95 | -0.23053870879 | 0.49445069 | 3,810.0000 | 0.421 |
| BCL2 | IGM | 0.074527253 | 0.95 | -0.30378174210 | 0.43253587 | 4,160.0000 | 0.695 |
| BCL2 | C3 | 0.425119911 | 0.95 | 0.06548475934 | 0.68701014 | 2,584.0860 | 0.019 |
| BCL2 | C4 | 0.285873788 | 0.95 | -0.09400090514 | 0.59308605 | 3,209.9973 | 0.126 |
| ESR | CRP | 0.824301815 | 0.95 | 0.65370784167 | 0.91516716 | 789.7633 | <0.001 |
| ESR | RF | 0.281517764 | 0.95 | -0.09869422917 | 0.59000647 | 3,229.5777 | 0.132 |
| ESR | CCP | 0.029847426 | 0.95 | -0.34388327335 | 0.39541511 | 4,360.8358 | 0.876 |
| ESR | IGA | 0.400845770 | 0.95 | 0.03629398350 | 0.67124325 | 2,693.1983 | 0.028 |
| ESR | IGG | 0.624012497 | 0.95 | 0.33033423498 | 0.80753271 | 1,690.0638 | <0.001 |
| ESR | IGM | 0.260821201 | 0.95 | -0.12076465290 | 0.57525091 | 3,322.6087 | 0.164 |
| ESR | C3 | 0.317108558 | 0.95 | -0.05984556212 | 0.61490793 | 3,069.5970 | 0.088 |
| ESR | C4 | 0.018499958 | 0.95 | -0.35385543168 | 0.38579337 | 4,411.8427 | 0.923 |
| CRP | RF | 0.161290323 | 0.95 | -0.22188235996 | 0.50131235 | 3,770.0000 | 0.395 |
| CRP | CCP | 0.099977773 | 0.95 | -0.28032373029 | 0.45315158 | 4,045.5999 | 0.599 |
| CRP | IGA | 0.315051732 | 0.95 | -0.06212218960 | 0.61348484 | 3,078.8425 | 0.090 |
| CRP | IGG | 0.383759733 | 0.95 | 0.01611362540 | 0.65999658 | 2,770.0000 | 0.036 |
| CRP | IGM | -0.027808676 | 0.95 | -0.39369230836 | 0.34568116 | 4,620.0000 | 0.884 |
| CRP | C3 | 0.384812575 | 0.95 | 0.01734853962 | 0.66069319 | 2,765.2675 | 0.036 |
| CRP | C4 | 0.134804086 | 0.95 | -0.24747060953 | 0.48076282 | 3,889.0556 | 0.478 |
| RF | CCP | -0.054998908 | 0.95 | -0.41645933700 | 0.32147550 | 4,742.2201 | 0.773 |
| RF | IGA | 0.246523530 | 0.95 | -0.13579423424 | 0.56493650 | 3,386.8767 | 0.189 |
| RF | IGG | 0.460734149 | 0.95 | 0.10945631255 | 0.70970496 | 2,424.0000 | 0.010 |
| RF | IGM | 0.439822024 | 0.95 | 0.08346918844 | 0.69644125 | 2,518.0000 | 0.015 |
| RF | C3 | -0.144304716 | 0.95 | -0.48817791704 | 0.23835301 | 5,143.6497 | 0.447 |
| RF | C4 | -0.278075205 | 0.95 | -0.58756630806 | 0.10239143 | 5,744.9480 | 0.137 |
| CCP | IGA | 0.089076967 | 0.95 | -0.29042703940 | 0.44436760 | 4,094.5990 | 0.640 |
| CCP | IGG | 0.435315247 | 0.95 | 0.07793146859 | 0.69355964 | 2,538.2580 | 0.016 |
| CCP | IGM | 0.064350950 | 0.95 | -0.31303471363 | 0.42418663 | 4,205.7425 | 0.735 |
| CCP | C3 | -0.135963447 | 0.95 | -0.48167034129 | 0.24636161 | 5,106.1557 | 0.474 |
| CCP | C4 | -0.048394302 | 0.95 | -0.41097051596 | 0.32740074 | 4,712.5324 | 0.800 |
| IGA | IGG | 0.628546005 | 0.95 | 0.33696308810 | 0.81011214 | 1,669.6857 | <0.001 |
| IGA | IGM | 0.088552676 | 0.95 | -0.29091085041 | 0.44394339 | 4,096.9557 | 0.642 |
| IGA | C3 | 0.325167167 | 0.95 | -0.05088783445 | 0.62046495 | 3,033.3736 | 0.080 |
| IGA | C4 | -0.085905383 | 0.95 | -0.44179901047 | 0.29335079 | 4,881.1447 | 0.652 |
| IGG | IGM | 0.403781980 | 0.95 | 0.03979214552 | 0.67316351 | 2,680.0000 | 0.027 |
| IGG | C3 | 0.159225111 | 0.95 | -0.22389670623 | 0.49972371 | 3,779.2831 | 0.401 |
| IGG | C4 | -0.122771985 | 0.95 | -0.47130039188 | 0.25892126 | 5,046.8601 | 0.518 |
| IGM | C3 | -0.297517130 | 0.95 | -0.60127367083 | 0.08137255 | 5,832.3395 | 0.110 |
| IGM | C4 | -0.312166155 | 0.95 | -0.61148507259 | 0.06530953 | 5,898.1869 | 0.093 |
| C3 | C4 | 0.566298739 | 0.95 | 0.24840392943 | 0.77407016 | 1,949.4872 | 0.001 |


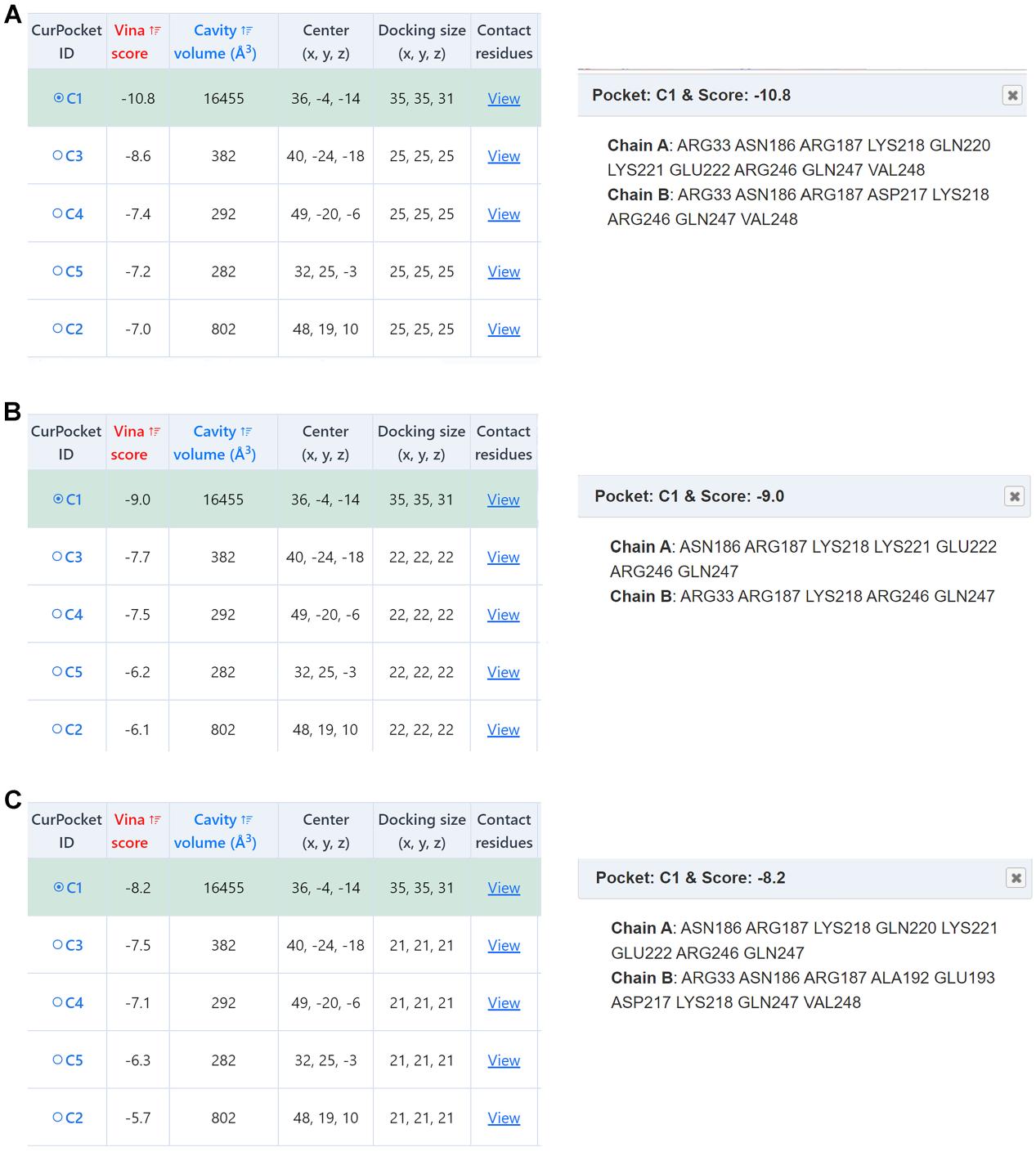


Supplementary Figure 1: Binding energies of molecular docking between active ingredients of XFC and p65. A: Binding energy of calycosin-7-O-beta-D-glucoside with P65. B: Binding energy of calycosin with P65. C: Binding energy of formononetin with P65.
